# Design, Implementation, and Analysis of an Assessment and Accreditation Model to Evaluate a Digital Competence Framework for Health Professionals: Mixed Methods Study

**DOI:** 10.2196/53462

**Published:** 2024-10-17

**Authors:** Francesc Saigí-Rubió, Teresa Romeu, Eulàlia Hernández Encuentra, Montse Guitert, Erik Andrés, Elisenda Reixach

**Affiliations:** 1 Faculty of Health Sciences Universitat Oberta de Catalunya Barcelona Spain; 2 Faculty of Psychology and Education Sciences Universitat Oberta de Catalunya Barcelona Spain; 3 Fundació TIC Salut i Social Generalitat de Catalunya Barcelona Spain

**Keywords:** eHealth literacy, eHealth competencies, digital health, competencies, eHealth, health literacy, digital technology, health care professionals, health care workers

## Abstract

**Background:**

Although digital health is essential for improving health care, its adoption remains slow due to the lack of literacy in this area. Therefore, it is crucial for health professionals to acquire digital skills and for a digital competence assessment and accreditation model to be implemented to make advances in this field.

**Objective:**

This study had two objectives: (1) to create a specific map of digital competences for health professionals and (2) to define and test a digital competence assessment and accreditation model for health professionals.

**Methods:**

We took an iterative mixed methods approach, which included a review of the gray literature and consultation with local experts. We used the arithmetic mean and SD in descriptive statistics, *P* values in hypothesis testing and subgroup comparisons, the greatest lower bound in test diagnosis, and the discrimination index in study instrument analysis.

**Results:**

The assessment model designed in accordance with the competence content defined in the map of digital competences and based on scenarios had excellent internal consistency overall (greatest lower bound=0.91). Although most study participants (110/122, 90.2%) reported an intermediate self-perceived digital competence level, we found that the vast majority would not attain a level-2 Accreditation of Competence in Information and Communication Technologies.

**Conclusions:**

Knowing the digital competence level of health professionals based on a defined competence framework should enable such professionals to be trained and updated to meet real needs in their specific professional contexts and, consequently, take full advantage of the potential of digital technologies. These results have informed the *Health Plan for Catalonia 2021-2025*, thus laying the foundations for creating and offering specific training to assess and certify the digital competence of such professionals.

## Introduction

### Background

The recent COVID-19 pandemic has highlighted the importance and potential of digital health in optimizing the quality, efficiency, and safety of health care [1-4]. Despite this, the adoption of digital tools and technologies in this field has been slow [5,6], and their full implementation in clinical practice has yet to occur [7]. Research has pointed to several factors as potential barriers, including technology, infrastructure, and financial resources [8,9]. However, it is the lack of digital health literacy that most commonly obstructs the implementation of digital health services [6]. Health professionals have been identified as a key factor in the digital transformation of health care [10]. Accordingly, they must be equipped with digital health competences, ranging from basic skills (eg, computer literacy) to more complex ones such as the ability to teach patients how to use technology and digital data sources safely and appropriately. Beyond informing patients about the availability and potential benefits of these technologies, physicians guide them on integrating these tools into their health care routines, playing a pivotal role in this process. For instance, they can guide patients on using portals for test results or mobile apps for medication adherence. However, for in-depth training on specific technologies, other professionals such as nurses or technical support staff may be better suited [2,6,9,11].

In 2016, a survey of 200 health professionals conducted by the European Health Parliament’s Digital Skills for Health Professionals (COMPDIG-Salut) Committee found that, in most cases, health professionals felt that they lacked the appropriate skills to cope with the digital revolution in their professional practice [12,13]. Today, there is still a need for accessible, structured, and comprehensive education that will enable future health professionals to make the best use of technology and harness its full potential in terms of quality of care [5,14,15].

Health professionals need to develop digital health competences to keep up with new technologies and ensure that they can provide high-quality patient care [2,5,7,16,17]. To this end, we must first map the specific digital competences needed in health care (to provide the right kind of digital education) [18] and then create a model for assessing and accrediting such competences. While there is a growing number of individual digital health competence frameworks and reviews that focus on specific health care professions or settings [2], there is a lack of standardization in the definition of digital health competences themselves, including discrepancies and overlap among available frameworks and their approach to categorization [7]. This implies a need to continually update the competences required in this field as well as the methods used to assess them [7].

The Professional Dialogue Forum of Catalonia (northeastern Spain), one of the most advanced regions in Europe in the use of digital health technologies [19-21], highlighted “the need to improve information and communication technology (ICT) competences to advance in the use of ICT and the design of telehealth services” as one of the 17 primary current and future challenges facing health professionals [22]. The COMPDIG-Salut project was launched in response to the identified needs, focusing on three aims: (1) defining a specific digital competence framework for health professionals, (2) creating a specific assessment and accreditation model for health professionals, and (3) designing actions to train and qualify health professionals in digital competences. Having determined the current digital competence level among Catalan health professionals [23], it is time to work toward achieving the aims of the COMPDIG-Salut project [12,24].

### Objectives

This study had two objectives: (1) to create a specific map of digital competences for health professionals and (2) to define and test a digital competence assessment and accreditation model for health professionals.

## Methods

### Study Design

The research presented in this paper is the result of collaboration between the TIC Salut Social Foundation (Information and Communication Technologies in Health and Social Care Foundation) and the Universitat Oberta de Catalunya in Spain. As this was an observational exploratory study focusing on the analysis of digital competences in health care, a mixed qualitative and quantitative methodology was used following an iterative approach, and questionnaires were designed for data collection.

### Specific Map of Digital Competences for Health Professionals

#### Narrative Review

A narrative review [25,26] was conducted to explore a broad range of existing digital competence frameworks in the field of health care and identify commonalities and strengths among the specific digital competences they included. We used the Google Scholar database to perform an iterative search for relevant frameworks based on a combination of search terms or keywords and appropriate Boolean operators.

Specifically, we searched for “digital competence framework” OR “digital capabilities framework” and “health professionals” within a search period spanning January 1, 2017, to October 13, 2021. The inclusion of potential frameworks of interest was based on the research team’s knowledge and expertise on the topic. Only publications in English and Spanish were considered.

#### Inclusion and Exclusion Criteria

Deciding which frameworks to include in our review required careful and deliberate consideration to avoid bias and ensure valid results. To this end, we established explicit inclusion and exclusion criteria to select complete frameworks (eg, they needed to comprise digital competences specific to health care). Expert synthesis, discussion, and agreement among ≥2 reviewers were required to select frameworks for inclusion in the narrative review and ensure a consistent selection process.

**Figure 1:**
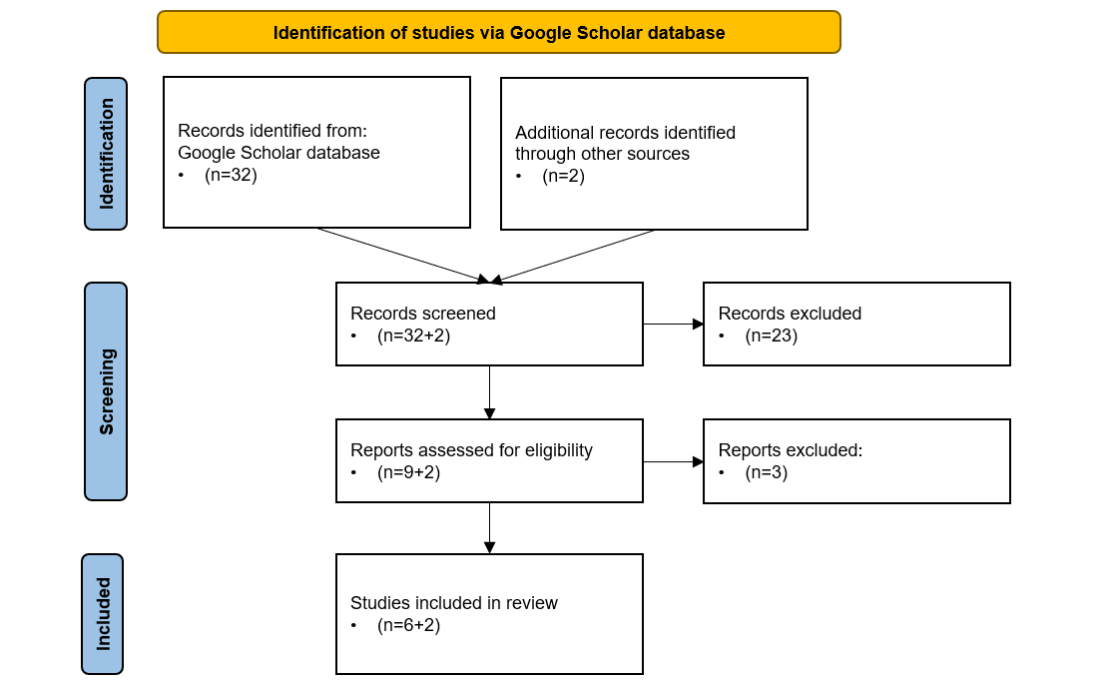
Flowchart of the literature search.

To reduce selection bias and facilitate comparisons between frameworks, we ascertained the specific actions in thematic areas, the number of defined competences, the levels of achievement contained in them, and whether they distinguished between health professions. We also looked for similarities between them and the European Digital Competence Framework for Citizens (DigComp) [27]. From the 32 results of the initial search, 9 (28%) frameworks were selected based on our inclusion and exclusion criteria. Of these 9 frameworks, 6 (67%) were selected for full-text analysis [28-33]. In total, 2 additional reference frameworks were included because of their relevance to mapping the digital competences of health professionals in Catalonia. These were the framework of digital competences for health professionals developed by the working group of challenge 4 of the Professional Dialogue Forum [22] and the Accreditation of Competence in Information and Communication Technologies (ACTIC) [34], the government of Catalonia’s framework for digital competence accreditation for citizens, which is currently in line with DigComp [27]. The latter was included because our proposed framework is closely related to it (Multimedia Appendix 1). The overview flowchart is shown in Figure 1.

#### Development of the Digital Competence Framework for Health Professionals and Area Specification

To develop the Digital Competence Framework for Health Professionals, we needed to specify the digital competences required of health professionals to manage digital health effectively, critically, and responsibly. Among the various initiatives led by the European Commission to improve people’s digital literacy is DigComp, which is “an umbrella or meta-framework for current frameworks, initiatives, curricula, and certifications” [28]. Using DigComp as a reference, we mapped the 6 selected frameworks, projects, and studies to establish relationships and connections between the identified keywords and the most relevant competences. We ordered the competences by similarity to reveal thematic areas and common content (Multimedia Appendix 2).

Axial coding was then applied to this content distribution to define areas, competences, and indicators [35]. Coding was performed using the ATLAS.ti software (version 22; ATLAS.ti Scientific Software Development GmbH; Multimedia Appendix 3).

These areas and competences were validated by a panel of 12 experts using a web-based questionnaire developed by the researchers. Considering the validation criteria (wording, consistency, applicability, and relevance), the experts assessed the clarity and precision of the labeling and the description of the competence areas, validated them or suggested changes, and answered open-ended questions on each item. The experts’ feedback was used to refine some of the definitions (Multimedia Appendix 4).

Indicators were then defined for each of the competences to determine which aspects should be assessed for all health professionals. Indicators are characteristics that can be observed through specific tests by either predefined measures or other qualitative information. Finally, 21 professionals of various profiles from the working group of challenge 4 of the Professional Dialogue Forum validated the framework of competence areas and indicators through a web-based questionnaire (Multimedia Appendix 5).

### Digital Competence Assessment and Accreditation Model for Health Professionals

#### Test Creation and Administration

Having developed the Digital Competence Framework for Health Professionals, the next step was to create an assessment and accreditation model. Given the variation in health professionals’ roles, we could not reduce this process to a one-size-fits-all test. So, to ensure that it would be relevant to each health professional’s activities and duties, 4 professional profiles were defined to account for most cases (Table 1).

**Table 1:** Professional profiles and their descriptions.

Profile code	Profile	Description
P1	Direct patient care	Professionals who spend >70% of their workday providing direct patient care or services (eg, physicians, nurses, occupational therapists, speech therapists, optometrists, opticians, dental hygienists, and pharmacists)
P2	Indirect patient care	Professionals who spend >70% of their workday providing health care support services (eg, physicians working in biological diagnostic and pathological anatomy services, specialist biologists, specialist physicists, specialist chemists, pharmacists, and dental prosthetists)
P3	Innovation, research, and teaching	Professionals who spend >70% of their workday providing innovation, research, or teaching services (eg, researchers and innovation specialists)
P4	Management	Professionals who spend >70% of their workday managing centers, organizations, departments, services, or teams (eg, executives and middle managers)

The test questions in our assessment and accreditation model had to be linked to definitions of observable behaviors that could be put into practice in different professional settings within the Catalan health care environment. Observable behaviors are understood as practices or actions performed by health professionals as part of their work (eg, finding clinical information in databases, communicating and collaborating remotely with teams or patients, using information management tools, and creating content). As the point of the assessment was to determine respondents’ level of digital competence, we considered it appropriate to use assessment scenarios that would present them with challenges that they would need to overcome. Their attempts to deal with situations similar to those in the real world and provide the best possible digital response to the proposed challenges would provide greater insights into their competence level for each indicator. It would also allow respondents to put into practice other skills, such as problem-solving, critical thinking, and the analysis and responsible use of information and communications technology [23].

The test for the 4 professional profiles included a set of 2 cases (scenarios) with a total of 28 questions to be answered within 60 minutes. The test was contextualized for each of the 4 profiles: P1, P2, P3, and P4. The maximum score was 30 points (26 questions counted for a maximum of 1 point each, and 2 questions counted for a maximum of 2 points each). Wrong answers on the multiple-choice questions were scored negatively (−0.2 points). A minimum score of 70% (21/30) was required to pass the test. Participants who scored <21 points were categorized as “suggested level not achieved,” and those who scored between 21 and 30 points were categorized as “suggested level achieved.”

#### Implementation and Analysis of the Assessment and Accreditation Model

To analyze the proposed assessment and accreditation model for our competence framework and identify areas for improvement (eg, time allotted, number of test questions, types of test questions, professional profiles, real-life situational approach by profile, assessment scenarios, and suitability of the cases and challenges presented) and validate the proposed level test (ie, to determine whether the proposed level was appropriate), we conducted a pilot study involving a web-based test with legally recognized health professionals [36] and health care social workers employed in Catalonia who reported an intermediate or advanced self-perceived digital competence level.

The web-based test consisted of 3 activities: a level test based on the exam required to obtain the ACTIC 2—intermediate level certificate [23] (activity 1); the test developed for our proposed assessment and accreditation model, as described in the previous section (activity 2); and a feedback questionnaire to understand participants’ opinions on the proposed assessment and accreditation model and other aspects of the pilot test (activity 3). The estimated time to complete the 3 activities was 90 minutes. The activities were to be done consecutively and in the specified order.

The ACTIC 2—intermediate level test (activity 1) was used to determine the participants’ baseline digital competence level and compare their scores with the results of the proposed assessment and accreditation test. Participants were categorized into 1 of the following 3 groups based on their scores: beginner (0-9.9), basic (10-24.9), and intermediate (25-35).

In relation to the test developed for our proposed assessment and accreditation model (activity 2), Table 2 shows all the variables involved in the pilot study.

**Table 2:** Variables for which data were collected from participants during the study.

Descriptor	Collection method
Name and surname	Forms (Microsoft Corp) questionnaire
Email address	Microsoft Forms questionnaire
Health profession	Microsoft Forms questionnaire
Self-perceived digital competence level	Microsoft Forms questionnaire
Experience related to digital competence training	Microsoft Forms questionnaire
Official certification in digital competences	Microsoft Forms questionnaire
Professional profile (P1, P2, P3, or P4)	Microsoft Forms questionnaire
Score achieved in activity 1	Moodle (Moodle HQ) questionnaire
Score achieved in activity 2	Moodle (Moodle HQ) questionnaire
Feedback questionnaire	Moodle (Moodle HQ) questionnaire

The feedback questionnaire (activity 3) consisted of 9 questions, 3 (33%) of which were open-ended and 1 (11%) of which asked for the participants’ self-perceived level in each of the digital competences defined for health professionals.

#### Data Collection

To focus the scope of the study, the Catalan Ministry of Health asked relevant professional associations to invite members whom they felt could meet the study’s inclusion criteria to volunteer as participants. Volunteer participants were recruited using a Microsoft Forms (Microsoft Corp) questionnaire (Multimedia Appendix 6). After informing them of the purpose of the study, their personal and professional details were collected. If they met the inclusion criteria, they were enrolled in the study and given credentials to access Moodle (Moodle HQ) for the web-based test.

Although the study was scheduled to remain open for 30 days, from March 3 to 31, 2022, it was ultimately extended to April 14, 2022, to increase the response rate. During this period, 2 emails were sent to all candidates to remind them of the study’s end date (or to inform them of the extension) and the remaining activities to be completed.

After this period, the test results were reported in accordance with “Good practice in the conduct and reporting of survey research” [31] and the General Data Protection Regulation, where applicable.

### Statistical Analysis

When designing the study, we calculated the minimum sample size to ensure significant results with a 10% error rate, the maximum allowed in research studies [37]. On the basis of the results of an exploratory study [23] and the latest available report on the population of health professionals in Catalonia [22], the minimum sample size for the study was set at 168.

Descriptive statistics were performed for professionals who had correctly completed activities 1 and 2, with results presented as absolute and relative frequencies. Arithmetic means and SDs were used for comparative analysis of subgroups according to sociodemographic and professional characteristics, and *P* values were used for hypothesis testing.

The reliability of activity 2 was analyzed by measuring the consistency of its items. In addition, the level of discrimination of the items in relation to the advanced level was evaluated. The arithmetic mean and SD were used in descriptive statistics; *P* values, Bonferroni-adjusted *P* values, and Holm-adjusted *P* values were used in hypothesis testing and subgroup comparisons; and the greatest lower bound (GLB) was chosen to diagnose the test given the lack of homogeneity of the scoring scale, as was done in the exploratory study [23].

The study instrument was analyzed using the participants’ scores and the discrimination index (DI) [38]. The DI measures how well an item could discriminate between high-scoring participants (ie, those with strong digital competences) and low-scoring participants in activity 2. DI values between 0 and 0.2 indicate that the item is not discriminating, and negative values imply an inverse relationship between the score on that item and the total score.

Finally, for activity 3, the numerical variables were presented as arithmetic means and SDs, whereas the categorical variables were presented as absolute and relative frequencies.

All responses were analyzed using the R statistical software (version 4.2.0; R Foundation for Statistical Computing). Responses to the open-ended questions (questions 5 and 7) in the feedback questionnaire (activity 3) were analyzed using quantitative content analysis to group them into limited categories [39].

### Ethical Considerations

No ethics approval was required due to the type and nature of the study as the Catalan Department of Health is responsible for formulating the general criteria for health planning, setting the objectives, and the levels to be achieved in the topics that are included in the *Health Plan for Catalonia* [40]. All participants were informed about the study’s purposes and that their participation was voluntary. Data protection treatment was informed to the participant and before accessing the survey, participants had to provide acceptance.

## Results

### Specific Map of Digital Competences for Health Professionals

Multimedia Appendix 7 shows the specific map of digital competences for health professionals validated by the 21 individuals (of different profiles) in the working group of challenge 4 of the Professional Dialogue Forum.

### Implementation and Analysis of the Digital Competence Assessment and Accreditation Model for Health Professionals

#### Recruitment

During the study period, we recorded a total of 398 visits to the Microsoft Forms recruitment questionnaire. Of the 398 visitors, 377 (94.7%) agreed to participate and gave their consent for the TIC Salut Social Foundation to process their personal data. After excluding participants who did not meet the inclusion criteria and removing repeated registrations, the total recruited sample comprised 372 participants, who were given access to Moodle to start the 3 programmed activities. A total of 49.5% (184/372) of the participants logged into Moodle and started the designed activities. In the end, 176 professionals completed activity 1 correctly, of whom 122 (69.3%) also completed activity 2 correctly.

For the purposes of this study, we considered a result as valid when the test was completed, allowing for a maximum of 3 consecutive questions with no blank answers.

#### Sample Description

The sample consisted mainly of health professionals with a direct patient care profile (P1; 95/122, 77.9%), followed by those with a management profile (P4; 13/122, 10.7%); an innovation, research, and teaching profile (P3; 8/122, 6.6%); and an indirect patient care profile (P2; 6/122, 4.9%; Table 3).

**Table 3:** Descriptive statistics for participants who correctly completed activities 1 and 2 (N=122).

Descriptor			Participants, n (%)
**Profile**			
	P1—direct patient care	95 (77.9)	
	P2—indirect patient care	6 (4.9)	
	P3—innovation, research, and teaching	8 (6.6)	
	P4—management	13 (10.7)	
**Health profession**			
	Specialist biologist	1 (0.8)	
	Dietician or nutritionist	1 (0.8)	
	Pharmacist	21 (17.2)	
	Specialist physical chemist	0 (0)	
	Physical therapist	17 (13.9)	
	Dental hygienist	7 (5.7)	
	Nurse	37 (30.3)	
	Speech therapist	7 (5.7)	
	Physician	4 (3.3)	
	Dentist	0 (0)	
	Optometrist/optician	6 (4.9)	
	Podiatrist	5 (4.1)	
	Dental prosthetist	1 (0.8)	
	Clinical or general psychologist	4 (3.3)	
	Occupational therapist	9 (7.4)	
	Health care social worker	2 (1.6)	
**Self-perceived digital competence level**			
	Intermediate	110 (90.2)	
	Advanced	12 (9.8)	
**ACTIC^a^ certification**			
	No certification	101 (82.8)	
	ACTIC 1	1 (0.8)	
	ACTIC 2	14 (11.5)	
	ACTIC 3	6 (4.9)	
**Experience at digital health events**			
	No experience	107 (87.7)	
	Speaker or trainer	11 (9)	
	Organizer	2 (1.6)	
	Both	2 (1.6)	

^a^ACTIC: Accreditation of Competence in Information and Communication Technologies.

Responses were received from professionals in all health professions, with the exception of specialist physical chemists and dentists. Nurses represented the largest proportion of the sample (37/122, 30.3%), followed by pharmacists (21/122, 17.2%) and physical therapists (17/122, 13.9%). According to the distribution of health care professions in Catalonia, a representative sample of nurses was obtained; however, this was not the case for physicians [41].

The vast majority of the sample (110/122, 90.2%) reported an intermediate self-perceived digital competence level, with only 9.8% (12/122) of participants considering themselves advanced. Overall, 82.8% (101/122) had no ACTIC certification, 11.5% (14/122) had an ACTIC 2 (intermediate) certification, 4.9% (6/122) had an ACTIC 3 (advanced) certification, and 0.8% (1/122) had an ACTIC 1 (basic) certification.

#### Activity 1: ACTIC 2—Intermediate Level Test (Baseline Level)

The mean total score for activity 1 was 24.3 (SD 4.1) points, which was significantly lower (*P*=.03) than the cutoff score for “Intermediate” (25 points). Of the 122 participants, 59 (48.4%) scored between 10 and 24.9 points (basic), and 63 (51.6%) scored ≥25 points (intermediate). On average, the sample took 12.2 (SD 3.8) minutes to complete the baseline level test. There were no outliers in the times recorded (Table 4).

**Table 4:** Summary of activity 1 results (N=122).

Descriptor		Values
Score, mean (SD)		24.3^a^ (4.1)
**Score range (points), n (%)**		
	<10 (beginner)	0 (0)
	10-24.9 (basic)	59 (48.4)
	≥25 (intermediate)	63 (51.6)
Minutes taken to complete the activity, mean (SD)		12.2 (3.8)

^a^Scores significantly below 25 points (*P*=.03).

The scores followed a normal distribution, with a mode of 25 points. Only 0.8% (1/122) of the participants obtained the maximum possible score on the level test (Multimedia Appendix 8).

Subgroups were compared to determine whether there were any significant differences in the overall scores. It was found that participants who had experience at digital health events scored significantly higher than those who did not (*P*=.03).

The scores for each subgroup were also compared to determine which subgroups scored significantly below 25 points in activity 1 (ie, did not reach intermediate level) and which scored significantly above 25 points (ie, did reach intermediate level). Several subgroups were found to have scored significantly below 25 points, including P1 (direct patient care; *P*=.04), Dental hygienist (*P*=.01), Intermediate level self-perception (*P*=.03), no ACTIC certification or ACTIC 1 (*P*=.02), no experience in digital health events (*P*=.02). Some subgroups scored significantly above 25 points, particularly the group of participants who reported ACTIC 3 certification (*P*=.40). P2 (indirect patient care) professionals and professionals with experience at digital health events scored significantly above 25 points, but this difference did not remain significant after correcting for multiple testing using the Bonferroni and Holm methods (Table 5).

**Table 5:** Overall scores and subgroup comparisons according to participant characteristics for activity 1.

Subgroup			Overall score, mean (SD)		Group comparison^a^							Intermediate level check											
					Unadjusted *P* value		Bonferroni*-*adjusted *P* value		Holm-adjusted *P* value		<25 points^b^								≥25 points^c^				
											Unadjusted *P* value			Bonferroni-adjusted *P* value		Holm-adjusted *P* value		Unadjusted *P* value			Bonferroni-adjusted *P* value		Holm-adjusted *P* value
**Profile**					.23		>.99		.52														
	P1—direct patient care (n=95)	24.1 (4.0)								*.01* ^d^			*.04*		*.04*		.99			>.99		>.99	
	P2—indirect patient care (n=6)	27.6 (2.3)								.98			>.99		>.99		*.02*			.08		.08	
	P3—innovation, research, and teaching (n=8)	24.7 (4.3)								.42			>.99		>.99		.58			>.99		>.99	
	P4—management (n=13)	24.5 (5.2)								.37			>.99		>.99		.63			>.99		>.99	
**Health profession^e^**					.60		>.99		.60														
	Nurse (n=37)	24.39 (4.17)								.19			>.99		>.99		.81			>.99		>.99	
	Pharmacist (n=21)	25.31 (3.31)								.66			>.99		>.99		.33			>.99		>.99	
	Physical therapist (n=17)	24.24 (5.28)								.28			>.99		>.99		.72			>.99		>.99	
	Other (n=44)^f^	23.83 (3.93)								*.02* ^d^			.25		.23		.98			>.99		>.99	
	Specialist biologist (n=1)	—^g^								—			—		—		—			—		—	
	Dietician or nutritionist (n=1)	—								—			—		—		—			—		—	
	Dental hygienist (n=7)	20.14 (2.56)								*.001* ^h^			*.01*		*.01*		>99			>.99		*>.99*	
	Speech therapist (n=7)	23.43 (4.75)								.21			>.99		>.99		.79			>.99		>.99	
	Physician (n=4)	22.75 (5.74)								.25			>.99		>.99		.76			>.99		>.99	
	Optometrist/optician (n=6)	24.00 (4.12)								.29			>.99		>.99		.71			>.99		>.99	
	Podiatrist (n=5)	24.10 (2.38)								.22			>.99		>.99		.78			>.99		>.99	
	Dental prosthetist (n=1)	—								—			—		—		—			—		—	
	General health or clinical psychologist (n=4)	23.89 (4.23)								.32			>.99		>.99		.68			>.99		>.99	
	Occupational therapist (n=9)	25.28 (3.16)								.60			>.99		>.99		.40			>.99		>.99	
	Health care social worker (n=2)	29.00 (0.71)								—			—		—		—			—		—	
**Self-perceived digital competence level**					.05		.26		.20														
	Intermediate (n=110)	24.12 (4.18)								*.02* ^d^			*.03* ^d^		*.03* ^d^		.99			>.99		.99	
	Advanced (n=12)	26.04 (2.85)								.88			>.99		.88		.12			.23		.23	
**ACTIC^i^ certification^e^**					.17		.87		.52														
	No certification/ACTIC 1 (n=102)	24.00 (4.14)								*.008* ^h^			*.02* ^d^		*.02* ^d^		.99			>.99		.99	
	ACTIC 2 (n=14)	25.04 (3.74)								.51			>.99		>.99		.49			>.99		.97	
	ACTIC 3 (n=6)	27.92 (2.27)								.99			>.99		>.99		*.01* ^d^			*.04* ^d^		*.04* ^d^	
**Experience at digital health events^e^**					*.006* ^j^		*.03* ^f^		*.03* ^f^														
	No experience (n=107)	24.03 (4.21)								*.01* ^d^			*.02* ^d^		*.02* ^d^		.99			>.99		.99	
	Some experience^k^ (n=15)	26.30 (2.50)								.97			>.99		0.97		*.03* ^d^			.06		.06	

^a^2-tailed *t* test for comparisons between 2 samples and 2-tailed ANOVA for comparisons among >2 samples.

^b^1-tailed *t* test with a defined overall score threshold of <25 points.

^c^1-tailed *t* test with a defined overall score threshold of ≥25 points.

^d^Significantly less or significantly more than 25 points at *P*<.05.

^e^Subgroups with too small a subsample size (n<6) were excluded from the analysis.

^f^Significant differences at *P*<.05.

^g^Subgroups with a subsample size of n=1 were excluded from the analysis.

^h^Significantly less or significantly more than 25 points at *P*<.01.

^i^ACTIC: Accreditation of Competence in Information and Communication Technologies.

^j^Significant differences at *P*<.01.

^k^Includes experience as a speaker, trainer, or organizer.

#### Activity 2: Proposed Assessment and Accreditation Test for the Map of Digital Competences for Health Professionals

##### Overview

The mean score for activity 2 was 18.5 (SD 3.7) points, which was very significantly lower (*P*<.001) than the cutoff score for passing the test (21 points). Of the 122 participants, 89 (73%) scored <21 points (did not pass), and 33 (27%) scored ≥21 points (passed). On average, the sample took 34.4 (SD 11.4) minutes to complete the test. There were no outliers in the times recorded (Table 6).

**Table 6:** Summary of activity 2 results (N=122).

Descriptor		Values
Score, mean (SD)		18.5^a^ (3.7)
**Score range (points), n (%)**		
	<21 (did not pass)	89 (73)
	≥21 (passed)	33 (27)
Minutes taken to complete the activity, mean (SD)		34.4 (11.4)

^a^Scores significantly below 21 points (*P*<.001).

An internal consistency analysis of activity 2 for P1 showed excellent internal consistency overall (GLB=0.91). The internal consistency of activity 2 could not be determined for the remaining profiles because there were not enough participants in them (Multimedia Appendix 9).

For activity 2, subgroup comparisons revealed significant differences between health professions and between professionals with intermediate and advanced self-perceived competence levels. However, the comparisons did not remain significant after applying the Bonferroni and Holm adjustments for multiple comparisons.

Some subgroups did not score significantly below 21 points, including P2 professionals (*P*=.83), nurses (*P*=.01), physical therapists (*P=*.22), speech therapists (*P*=.18), physicians (*P*>.99), podiatrists (*P*=.37), occupational therapists (*P*=.78), professionals with an advanced self-perceived competence level (*P=*.16), professionals with ACTIC 3 certification (*P*>.99), and professionals with experience at digital health events (*P*=.23). In some cases, the lack of significant level determination was likely due to the small size of the subgroup (Table 7).

**Table 7:** Overall scores and subgroup comparisons according to participant characteristics for activity 2.

Subgroup			Overall score, mean (SD)		Group comparison^a^							Intermediate level check											
					Unadjusted *P* value		Bonferroni-adjusted *P* value		Holm-adjusted *P* value		<21 points^b^								≥21 points^c^				
											Unadjusted *P* value			Bonferroni-adjusted *P* value		Holm-adjusted *P* value		Unadjusted *P* value			Bonferroni-adjusted *P* value		Holm-adjusted *P* value
**Profile**					.24		>.99		.35														
	P1—direct patient care (n=95)	18.60 (3.71)								*<.001* ^d^			*<.001* ^d^		*<.001* ^d^		>.99			>.99		>.99	
	P2—indirect patient care (n=6)	20.14 (2.37)								.21			.83		.21		.79			>.99		>.99	
	P3—innovation, research, and teaching (n=8)	18.51 (1.77)								*.003* ^d^			*.01* ^e^		*.006* ^d^		>.99			>.99		>.99	
	P4—management (n=13)	16.74 (4.43)								*.002* ^d^			*.008* ^d^		*.006* ^d^		>.99			>.99		>.99	
**Health profession^f^**					*.03* ^g^		.14		.11														
	Nurse (n=37)	19.59 (3.59)								*.01* ^e^			.11		.07		.99			>.99		>.99	
	Pharmacist (n=21)	18.59 (3.07)								*<.001* ^d^			*.01* ^e^		*.007* ^d^		>.99			>.99		>.99	
	Physical therapist (n=17)	19.16 (3.47)								*.02* ^e^			.22		.10		.98			>.99		>.99	
	Other (n=44)^g^	17.28 (3.87)								*<.001* ^d^			*<.001* ^d^		*<.001* ^d^		>.99			>.99		>.99	
	Specialist biologist (n=1)	—^h^								—			—		—		—			—		—	
	Dietician or nutritionist (n=1)	—								—			—		—		—			—		—	
	Dental hygienist (n=7)	12.47 (1.70)								*<.001* ^d^			*<.001* ^d^		*<.001* ^d^		>.99			>.99		>.99	
	Speech therapist (n=7)	16.87 (3.92)								*.02* ^e^			.18		.10		.98			>.99		>.99	
	Physician (n=4)	19.52 (3.77)								.24			>.99		.32		.76			>.99		>.99	
	Optometrist/optician (n=6)	15.76 (1.80)								*<.001* ^d^			*.005* ^d^		*.004* ^d^		>.99			>.99		>.99	
	Podiatrist (n=5)	15.99 (4.49)								*.03* ^f^			.37		.14		.97			>.99		>.99	
	Dental prosthetist (n=1)	—								—			—		—		—			—		—	
	General health or clinical psychologist (n=4)	19.13 (3.11)								.16			>.99		.32		.84			>.99		>.99	
	Occupational therapist (n=9)	19.51 (2.73)								.07			.78		.21		.93			>.99		>.99	
	Health care social worker (n=2)	21.67 (0.23)								—			—		—		—			—		—	
**Self-perceived digital competence level**					*.02* ^g^		.09		.09														
	Intermediate (n=110)	18.29 (3.78)								*<.001* ^d^			*<.001* ^d^		*<.001* ^d^		>.99			>.99		>.99	
	Advanced (n=12)	20.1 (2.06)								.08			.16		.08		.92			>.99		>.99	
**ACTIC^i^ certification^f^**					.12		.59		.35														
	No certification/ACTIC 1 (n=102)	18.17 (3.82)								*<.001* ^d^			*<.001* ^d^		*<.001* ^d^		>.99			>.99		>.99	
	ACTIC 2 (n=14)	19.31 (2.35)								*.008* ^d^			*.02* ^e^		*.02* ^e^		.99			>.99		>.99	
	ACTIC 3 (n=6)	21.55 (1.70)								.77			>.99		.77		.23			.70		.70	
**Experience at digital health events^f^**					.13		.66		.35														
	No experience (n=107)	18.28 (3.67)								*<.001* ^d^			*<.001* ^d^		*<.001* ^d^		>.99			>.99		>.99	
	Some experience^j^ (n=15)	19.83 (3.56)								.11			.23		.11		.89			>.99		>.99	

^a^2-tailed *t* test for comparisons between 2 samples and 2-tailed ANOVA for comparisons among >2 samples.

^b^1-tailed *t* test with a defined overall score threshold of <21 points.

^c^1-tailed *t* test with a defined overall score threshold of ≥21 points.

^d^Very significantly less than 21 points at *P*<.01.

^e^Significantly less than 21 points at *P*<.05.

^f^Subgroups with too small a subsample size (n<6) were excluded from the analysis.

^g^Significant differences at *P*<.05.

^h^Subgroups with a subsample size of n=1 were excluded from the analysis.

^i^ACTIC: Accreditation of Competence in Information and Communication Technologies.

^j^Includes experience as a speaker, trainer, or organizer.

##### Item Dimension (Instrument Diagnosis)

It should be noted that we performed this analysis only for activity 2 and P1 (direct patient care) as this subgroup was large enough for this type of analysis (n>40; Multimedia Appendix 10).

#### Correlation Between Participants’ Performance in Activity 1 and Activity 2

Regarding the correlation between participants’ performance in activity 1 and activity 2, there were 2 main groups: those who did not pass either activity 1 or activity 2 (49/122, 40.2%) and those who passed activity 1 but did not pass activity 2 (40/122, 32.8%) (Table 8).

**Table 8:** Participant classification according to performance in activity 1 and activity 2 (N=122).

Descriptor	Participants, n (%)
Passed activity 1 and passed activity 2	23 (18.9)
Passed activity 1 and did not pass activity 2	40 (32.8)
Did not pass activity 1 and passed activity 2	10 (8.2)
Did not pass activity 1 and did not pass activity 2	49 (40.2)

Of the 33 participants who passed activity 2, a total of 10 (30%) did not pass activity 1. Although these are isolated cases, they call into question the validity of the advanced level of activity 2 and need to be investigated further. No significant differences were found between this subgroup and the remaining professionals in the time taken to complete or in the number of blank answers in activity 1.

#### Activity 3: Feedback Questionnaire

A total of 94.3% (115/122) of the participants completed activity 3, although not all of them answered every question.

There were no significant differences in the ratings by profile (P1, P2, P3, and P4) or by health profession that would allow for robust conclusions to be drawn (Table 9).

**Table 9:** Results of the feedback questionnaire (N=115).

Question number and topic		Result
Question 1—level of difficulty of activity 2, mean (SD)		3.6 (0.7)
**Question 2—self-perceived level in activity 2, n (%)**		
	Basic	7 (6.1)
	Intermediate	49 (42.6)
	Advanced	59 (51.3)
Question 3—wording of the questions, mean (SD)		3.8 (0.9)
Question 4—appropriateness of the challenges, mean (SD)		3.2 (1.2)
Question 5—feedback on the challenges and scenarios		Open-ended question
Question 6—appropriateness of the profiles, mean (SD)		4.2 (0.8)
Question 7—feedback on the profiles		Open-ended question
Question 8—self-perceived level in each of the digital competences defined for health professionals		Multiple-choice question
Question 9—general feedback on the pilot study and the initiative		Open-ended question

When asked whether they would change any of the challenges or scenarios presented in activity 2 as part of our assessment and accreditation model (question 5), a considerable number of professionals (45/94, 48%) indicated that they did not fully identify with them (Multimedia Appendix 11).

When the responses were analyzed by health profession, we found that nurses and physicians generally identified with the challenges more than their counterparts in other health professions did (Multimedia Appendix 12). While 48% (11/23) of nurses and physicians gave positive feedback in this regard, only 31% (15/48) of respondents from other professions said that they adequately identified with the challenges and scenarios.

When asked whether they would change any of the profiles in our model (question 7), most participants (49/79, 62%) agreed that the profiles were accurately defined. Of the participants who did not fully agree, some questioned the workday percentage defined for categorization (eg, 70% direct patient care for P1). Others felt that there were hybrid profiles halfway between P1 and P4 or other profiles altogether or indeed that some profiles should be included in others (Multimedia Appendix 13).

It should be noted that the participants who indicated that they did not fully agree with the profiles were either from the private sector or working in pharmaceutical companies.

**Figure 2:**
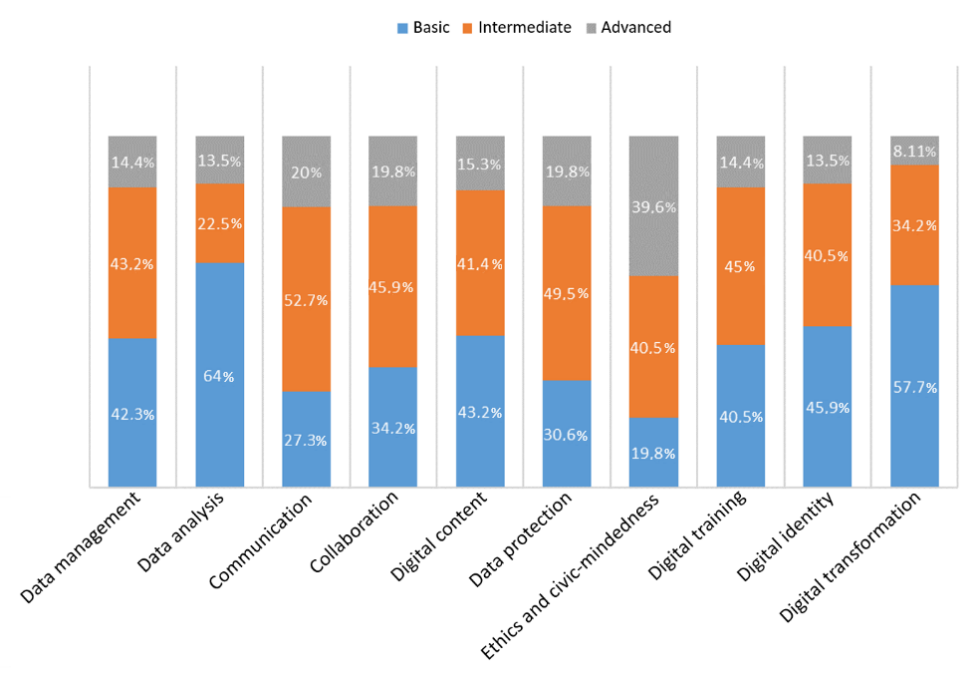
Participants’ self-perceived level in each of the digital competences defined for health professionals.

Regarding the participants’ self-perceived level in each of the 10 digital competences defined for health professionals in our framework (question 8), most reported having a basic competence level of data analysis and digital transformation (71/111, 64% and 64/111, 58%, respectively). The competence for which most professionals reported an advanced level was ethics and civic-mindedness (44/111, 40%; Figure 2).

## Discussion

### Frameworks and Competence Assessment Challenges

Digital health offers a valuable opportunity to make strides toward attaining the United Nations’ Sustainable Development Goal of universal health coverage by 2030 [42]. To that end, and given the gradual digitization of the health care sector, health professionals must have the appropriate competences regardless of their specific disciplines [2,5,16,17,43].

Furthermore, the constant evolution of new technologies requires the continuous updating of digital health competence. This entails reviewing both the relevant competences and the methods for properly assessing them [2]. Some discrepancies and overlap still exist among available frameworks, the methods used to conceptualize such frameworks, and the competences they include [2] and also among the health professionals at whom they are aimed [44,45]. The lack of a comprehensive framework applicable to all health professionals warrants the development of frameworks that include different health professionals.

Among the most recent frameworks is the Health Information Technology Competencies. Developed using an iterative method, it is the most complete framework aimed at a broad range of health professionals and medical specialties [30]. In Spain, and more specifically in the Basque Country (northern Spain), the Ikanos project was developed (2020) based on the European DigComp framework as a benchmark for the description of digital competences. Drawing on interviews with experts, the Ikanos framework focuses on profiles based initially on primary care professionals (medical and nursing staff) [29].

The COMPDIG-Salut project was launched for the purposes of designing a specific map of digital competences for health professionals and creating a specific digital competence assessment and accreditation model to enable health professionals to obtain a specific accreditation certificate. Taking an iterative approach, which included a review of the gray literature and consultation with local experts [2], we designed a map of digital competences, consisting of 10 competences under 4 competence areas: data access, management, and analysis; communication and collaboration; digital awareness; and professional development. The second and fourth of these areas have the highest number of competences (3 each). In short, the map includes competences related to essential skills for the digital management and analysis of health and social data and information and includes emerging technological innovation (ie, health apps, artificial intelligence, and autonomous decision support systems) [2,46]. Moreover, and given that digital health entails new methods of communication (teleconsultations and email), it is imperative for health professionals to know how to convey information in a precise, effective, and timely manner to patients, health professionals, and any collaborating parties [47-49]. Effective communication, collaboration, and teamwork are crucial in a health setting, so health professionals must also possess the ability to work effectively as members of interdisciplinary teams [2,50]. The creation and publication of health-related digital content is another basic competence that health professionals should have to ensure high-quality health care provision and help improve the communities they serve [31,51]. Finally, emphasis is placed on the importance of health professionals complying with the ethical principles and security criteria associated with the appropriate use of digital technologies [52] and also keeping abreast of the regulations on health and social data and information privacy, confidentiality, and protection [2,53,54]. However, we should bear in mind that the evolving nature of digital health and professional practice in the health field—which often outpaces research—requires constant, flexible development of professional competences [55]. Hence, some of the competences in the competence framework defined in the COMPDIG-Salut project may need to be adjusted to incorporate future digital trends, such as addressing challenges in the areas of cybersecurity and the use of artificial intelligence and robotics in professional practice. This implies that the methods for accurately assessing such competences may need to be updated.

### Digital Competence Assessment Model

Meanwhile, given the need to assess health professionals’ digital competence levels for the purposes of narrowing the digital gap and, by so doing, maintaining health service quality [11,45], a digital competence assessment model for health professionals was developed as part of the COMPDIG-Salut project. Drawing on the competence content defined in the project, the model was designed in accordance with ACTIC’s new scenario-based assessment model [23]. In this sense, the assessment model was adapted to the 4 distinct health sector profiles: professionals providing direct patient care; professionals providing indirect patient care; professionals providing innovation, research, or teaching services; and professionals who manage. This not only reflects the impact of the professional role on digital competence [56] but also, with the differentiation of the 4 profiles, facilitates the design of a specific training pathway for each role (Multimedia Appendix 14).

While the focus was on the type of health professional profile (P1, P2, P3, or P4), the most frequent professional categories into which participants fell were nurses, pharmacists, and physiotherapists. The internal consistency of the digital competence assessment model for health professionals in P1 was excellent overall, bearing in mind that it was a proposed assessment model (GLB=0.91). We found that 54% (15/28) of the questions discriminated between health professionals with an advanced level of digital competence and those with a lower level (DI≥0.20) and that 11% (3/28) of the questions were highly discriminating (DI>0.4). However, 7% (2/28) of the items had a negative DI, so these would need to be reviewed because a negative DI implies an inverse relationship between the level and the correct answer, meaning that the wording of the question may not have been sufficiently precise. However, as these values were close to 0, they could be regrouped with the nondifferentiating questions (DI=0-0.2). As the assessment model for the remaining profiles assessed the same competences as those in the P1 test, we expected the internal consistency and the degree of discrimination of the questions to be similar among the profiles. However, it was not possible to directly extrapolate the P1 results.

Most of the questions in the feedback questionnaire (activity 3) could not be robustly interpreted because they did not constitute a standard instrument that had been validated by another study. The values obtained in the Likert-type questions could not be robustly interpreted either because the values were not near the ends of the scale. Many health professionals felt that their roles and challenges were not adequately represented in the assessment model despite the professional profiles being well defined. This was especially the case among health professionals who made up the smallest groups (mostly nonmedical and nonnursing staff and those working in the private or pharmaceutical sectors). This result underscores the necessity of refining the assessment model to meet the diverse digital competence needs of health care professionals, emphasizing the potential requirement for more targeted measures tailored to specific groups, and acknowledging their potential cultural dependencies. Therefore, it would be necessary to either review the profile proposal for the competence assessment model or add scenarios and challenges to it for health professionals who were not reflected in the proposed situations. The sample size of some subgroups (physicians, health professionals with advanced digital competence, P2, P3, and P4) may have been too small to represent the population.

### Self-Perceived Digital Competence and Certification

Although most study participants (110/122, 90.2%) reported an intermediate self-perceived digital competence level and only 9.8% (12/122) of participants considered themselves advanced, we found that the vast majority would not attain ACTIC 2 (intermediate) certification [34]. This situation was similar to the one found in an exploratory study conducted in 2021 [23]. Consistent with the low score for the ACTIC 2 certification (activity 1), the total mean score for activity 2, which assessed the defined competence framework, was significantly lower than the defined threshold. In fact, 73% (89/122) of the sample did not pass the tests. Participants with ACTIC 3 certification significantly passed activity 1 but not activity 2. In fact, none of the subgroups studied showed a significant increase in activity 2. This shows that the assessment model is a useful tool as an approach to assessing the competence framework for health professionals, and the results strengthen those obtained in the exploratory study [23]. This also shows that health professionals still need training, that such training goes beyond that required for the main tools currently used, and that merely being exposed to digital media as consumers is insufficient in terms of acquiring the necessary digital skills. More and more faculties of medicine are incorporating digital health training into their programs of study [57,58], and there is an ever-increasing number of initiatives to meet that need [2], such as the Digital Capability Framework developed by Health Education England [59] or the free introduction to eHealth web-based course offered by the EU*US eHealth Work project [60]. The designed map of digital competences could serve as the basis of the knowledge and skills for digital literacy specific to health professionals, to which other competences or areas could be added in accordance with the particularities of the various health professions and medical specialties. This map of digital competences could also inform and contribute to the development of future digital health training programs for health workers at different stages of their professional careers.

### Strengths and Limitations

Our study provides a competence framework and a tool for assessing the digital competences of health professionals that is consistent with the certification available to citizens (ACTIC). It also yields results that strengthen those obtained in the previous exploratory study [23]. However, this study has several limitations. First, several weaknesses in the interpretation of findings from the review should be considered. While narrative reviews are often used in social science research for educational purposes, they may be biased and lack objectivity. That said, they can provide a unique perspective and identify knowledge gaps in the literature [61]. Although a considerable number of reference frameworks from gray literature were included, some may have been overlooked. Moreover, while performing thematic analysis, we found that some frameworks had either vague competence categories or overlaps among categories, which engendered differences of opinion during the classification process. However, the 4 reviewers drew on their experience to develop and clearly define the competence areas and assign the corresponding competences to them, first independently and then jointly through discussion-based agreement to reduce bias and classify the information in the most appropriate way possible. Although the reviewers tried to make the classification process as transparent and replicable as possible, it should be borne in mind that there could be other ways of interpreting and classifying the information and that the categorization might differ in the future with new advances in digital health. Second, while valid results were obtained for 122 participants, the minimum sample size set for the study was not reached (N=168). The platform used for the test (Moodle), the number of activities (3), and the time allotted (estimated at 1.5 hours) were almost certainly the reasons for poor participation in the study, especially given the high workload of the professional group in question. A study assessing both perceived and demonstrated eHealth literacy through a computer simulation of health-related internet tasks also revealed that evaluating demonstrated eHealth literacy via simulations is a challenging endeavor in terms of time [62]. When comparing the results of this study (using Moodle for the test) to those of the previous exploratory study (using Microsoft Forms for the test), it could be said that Moodle might have had an influence on a health professional’s decision not to take part in the study [23]. Third, the most highly represented health professionals in this study were those providing direct patient care (P1). Thus, many of the conclusions drawn from the study are limited to that profile. The poor participation of physicians (4/122, 3.3%) meant that they were underrepresented if compared to the 2017 professional population data for Catalonia [22]. As it fell to professional associations to inform their members about this study, the dissemination mechanisms they used are not precisely known. However, their impact was seemingly greater in the associations of nurses, pharmacists, and physiotherapists than in the associations of other health professions. Fourth, the absence of a detailed demographic analysis of the participant population limits our ability to provide a comprehensive understanding of sample characteristics. While demographic characterization could aid in contextualizing the results and their relationship to the simulation performance, such data were not collected in this study in accordance with the General Data Protection Regulation principle of “data minimization” [63]. Consequently, the lack of this analysis may restrict our ability to generalize findings to broader populations and fully comprehend the influence of demographic variables on study outcomes. Fifth, our findings enable us to estimate the prevalence of P1 as the most common profile; however, they do not provide a basis for drawing more specific conclusions. The 2022 report on health care professionals in Catalonia lacks profession categorization [22], and acquiring accurate and current data poses challenges attributable to factors such as professional mobility (eg, change of workplace, the coexistence of public and private sectors, and mobility between various institutions) and liberal professions. Subsequent research endeavors should prioritize the inclusion of all 4 profiles for a more comprehensive understanding. Sixth, the overrepresentation of health professionals with an intermediate self-perceived competence level compared to those considering themselves advanced also limited some of the study conclusions. We will probably need to review our definitions of the different self-perception levels because, perhaps due to the wording used, there was a greater perception of exigency than there really was (eg, advanced user: “I have attained the most advanced digital competences for transforming and innovating in today’s digital society.”). Moreover, the evaluation of digital health competences should take into account that, despite being notable, there is a moderate correlation between perceived and actual digital competences, as other studies looking at simulation scenarios in health care have shown [62,64]. Despite the considerable similarity between the latter simulation scenarios and those used in this study, previous research has focused on distinct demographic groups within the general population, such as individuals aged ≥50 years [62], or specific cohorts, such as patients with chronic illnesses [64], rather than health care professionals.

### Conclusions

Assessing the digital competence level of health professionals based on a defined competence framework should enable such professionals to be trained and updated to meet real needs in their specific professional contexts and, consequently, take full advantage of the potential of digital technologies [65]. The information and data gathered, together with the results of an exploratory study on competence levels conducted in 2021 [23], should be taken as the starting point for promoting relevant strategic policies and actions to ensure that the right resources and conditions are in place for good professional performance [15,66-69]. Faced with the need to improve the digital competence of health professionals working in Catalonia, these results have informed the *Health Plan for Catalonia 2021-2025* [40] and lay the foundations for designing and delivering specific training to first assess and then certify the digital competence of such professionals. Thus, the assessment model presented in this paper—designed in accordance with the competence content defined in the map of digital competences and based on scenarios—has the potential to be applied in diverse countries and languages with appropriate modifications to meet the specific needs and contexts of health care professionals. This might provide a preliminary perspective for assessing the competence levels and needs of health professionals in different health systems, although further evidence is needed to fully support this claim.

## Appendix

Multimedia Appendix 1 Reference frameworks selected for the analysis and comparative study.

Multimedia Appendix 2 Ordering of competences by thematic area for each framework.

Multimedia Appendix 3 Axial coding of content to define areas, competences, and indicators.

Multimedia Appendix 4 Web-based questionnaire 1.

Multimedia Appendix 5 Web-based questionnaire 2.

Multimedia Appendix 6 Information sheet and recruitment questionnaire.

Multimedia Appendix 7 Specific map of digital competences for health professionals.

Multimedia Appendix 8 Overall distribution of scores on Activity 1.

Multimedia Appendix 9 Overall distribution of scores in the proposed assessment and accreditation test for health professionals.

Multimedia Appendix 10 Discrimination indexes of the instrument items for profile P1.

Multimedia Appendix 11 Question 5: “Feedback on the profile–specific challenges and scenarios.”.

Multimedia Appendix 12 Question 5: “Feedback on the profile–specific challenges and scenarios by profession.”.

Multimedia Appendix 13 Question 7: “Feedback on the profiles.”.

Multimedia Appendix 14 Example scenario 1.

## Abbreviations

ACTIC: Accreditation of Competence in Information and Communication Technologies
COMPDIG-Salut: Digital Skills for Health Professionals
DI: discrimination index
DigComp: Digital Competence Framework for Citizens
GLB: greatest lower bound
