# Supplementary material for: Design, Implementation, and Analysis of an Assessment and Accreditation Model to Evaluate a Digital Competence Framework for Health Professionals: Mixed Methods Study
Source: JMIR Med Educ. 2024 Oct 17;10:e53462. doi: 10.2196/53462 (PMC11528169; doi:10.2196/53462)
Supplement: Multimedia Appendix 1 [file mededu_v10i1e53462_app1.docx]

**Appendix Table 1**. Reference frameworks selected for the analysis and comparative study.

| **Reference framework** | **Number of thematic areas** | **Number of competencies** | **Are there levels?** | **Are there profiles?** | **Is it similar to ACTIC?** | **Comments** |
| --- | --- | --- | --- | --- | --- | --- |
| [28] | 6 | 26 | NO | NO | YES |  |
| [29] | 5 | 19 | YES | YES | YES | It uses the same competencies as those for citizens and sets levels of achievement for each of them, adapted to the needs of the professional field. In other words, a professional must demonstrate a certain level in each competency.  It includes a proposal for defining each health professional profile using the same mechanism.  **It excludes competencies such as (1) online participation and innovation, and (2) creative use of technology (to be considered).**  **It includes specific health software in its indicators.** |
| [30] | 10 | 21 | YES | YES | NO | To define thematic areas and competencies, we used the definition of training associated with HITCOMP as a guide.  The tool has 1025 competencies by levels and professional profiles.  **It can be helpful for specifying indicators and competencies.**  It is very different from the ACTIC organization. |
| [31] | 6 | 52 | YES | YES | NO | The competence areas are: Plan, Run, Build, Enable, Manage, and Use. |
| [34] | 5 | 19 | NO | NO | YES | Focus on the competencies of ACTIC, as **it is necessary to follow the same line of definition** to facilitate management and assessment. |
| [22] | 7 | Not defined (a definition is included that could be a competency) | NO | NO | YES | We used this definition as a starting point for comparison with the other frameworks. |
| [32] | 6 | 30 | YES | NO | YES |  |
| [33] |  |  | NO | NO | NO | Profile of the health professional of the future. Eric Topol. |

**References**

22. Reunió plenària I Fòrum de diàleg professional. In: Departament de Salut [Internet]. [cited 27 Jul 2022]. Available: http://salutweb.gencat.cat/ca/el_departament/eixos-xiv-legislatura/forum-dialeg-professional/etapes/treballs-fase-anterior/i-forum-diagnostic/

28. Montero Delgado JA, Merino Alonso FJ, Monte Boquet E, Ávila de Tomás JF, Cepeda Díez JM. Competencias digitales clave de los profesionales sanitarios. Educación Médica. 2020;21: 338–344. doi:10.1016/j.edumed.2019.02.010

29. Test Ikanos de competencias digitales. [cited 26 Jul 2022]. Available: https://test.ikanos.eus/

30. EU*US eHealth Work Project. Health Information Technology Competencies. In: Health Information Technology Competencies (HITCOMP) [Internet]. 2020 [cited 26 May 2023]. Available: http://hitcomp.org/competencies/

31. JAseHN T7.1.3. eHealth competence model based on the previous framework (2018): Recommendations on a Common Framework for Mapping Health Professionals’ eHealth Competencies. Available: https://ec.europa.eu/research/participants/documents/downloadPublic?documentIds=080166e5bc2cac91&appId=PPGMS

32. NHS. A health and care digital capabilities framework [Framework]. 2018. Available: https://www.hee.nhs.uk/sites/default/files/documents/Digital%20Literacy%20Capability%20Framework%202018.pdf

33. The Topol Review — NHS Health Education England. In: The Topol Review — NHS Health Education England [Internet]. [cited 18 Sep 2023]. Available: https://topol.hee.nhs.uk/

34. What is ACTIC. [cited 26 Jul 2022]. Available: http://actic.gencat.cat/en/actic_informacio/actic_que_es_l_actic_/index.html
