# Supplementary material for: Design, Implementation, and Analysis of an Assessment and Accreditation Model to Evaluate a Digital Competence Framework for Health Professionals: Mixed Methods Study
Source: JMIR Med Educ. 2024 Oct 17;10:e53462. doi: 10.2196/53462 (PMC11528169; doi:10.2196/53462)
Supplement: Multimedia Appendix 2 [file mededu_v10i1e53462_app2.docx]

**Appendix Table 2**. Ordering of competencies by thematic area for each framework

| **Working Group 4 of the Forum for Professional Dialogue** |  | **ACTIC 2 – Intermediate level** | **Key digital skills for healthcare professionals** | | **A Health and Care Digital Capabilities Framework (UK)** | | **HITCOMP domain** | **HITCOMP – Advanced** |
| --- | --- | --- | --- | --- | --- | --- | --- | --- |
| Collaborate and interact remotely with healthcare stakeholders and promote networking among them. (Collaboration and participation) |  | 2.2 Collaborate with others using digital technologies | 5. Collaborative networking with healthcare teams | 5.1. Be familiar with and know how to use digital tools based on cloud technology that facilitate remote collaboration and teamwork. | Communication, Collaboration and Participation | a. The ability to use a wide range of digital technologies to communicate with people and to understand the different nature, purpose, and function of different methods of digital communication, acting accordingly and appropriately. |  |  |
|  |  |  |  | 5.2. Identify and make the most of web–based collaborative health networks and contribute to them appropriately. |  | b. The ability to use digital technologies to communicate respectfully and appropriately with all people and to recognize one’s responsibility to not engage in or allow others to engage in inappropriate, irresponsible, offensive, or harmful communication activities. |  |  |
|  |  |  |  | 5.3. Connect, converse, share knowledge, and collaborate with other professionals with common goals remotely over the Internet. |  | c. The ability to work collaboratively with others using digital technologies and tools to produce shared outcomes to meet shared goals. |  |  |
| Understand and critically use the forums and channels available for digital interaction. (Communication and digital reputation) | Communication and collaboration | 2.1 Interacts with and shares digital information and content. | 3. Healthcare communication 2.0 | 3.1 Properly manage your digital identity and reputation in healthcare settings. |  | d. The ability to participate actively in and across digital networks. | Direct Patient Care, Administration, Informatics | Ensure patients, caregivers and family know how to access information to inform their health and care, and where they can go for help and support |
|  |  | 2.2 Collaborates with others using digital technologies. |  | 3.2. Be familiar with the professional communities and networks that may be useful in healthcare. |  | e. The ability to demonstrate and champion ethical, positive, sensitive, and appropriate attitudes and behaviors in communicating, collaborating and participating with anybody and everybody. |  |  |
|  | Change management | 2.3 Gets involved in social and cultural change through the digital society. |  | 3.3. Appropriately use the new channels and languages of digital communication in healthcare, properly manage your digital identity, and demonstrate appropriate behavior in digital contexts (cyberethics, cybersecurity, privacy, and respect for data protection regulations). |  |  |  |  |
|  |  |  |  | 3.4. Connect, interact, and converse through digital platforms and tools used in healthcare settings. |  |  |  |  |
|  |  |  |  | 3.5. Use the Internet to publish and disseminate valuable digital health content that can attract the attention of the intended audience. |  |  |  |  |
| Facilitate process–based solution and product development environments and models in which ICTs become enablers for research and change. (Research, creation, and innovation) | Content creation, programming, and object fabrication | 3.1 Creates and edits digital content. | 4. Digital content creation in science and health | 4.1. Know what types of digital content exist and the tools available to create them. | Creation, Innovation and Research | a. The ability to create new digital resources and/or curate existing ones working individually or in collaboration with others. | Direct Patient Care | Construct and utilize clinical pathways and guidelines to improve clinical practice |
|  |  | 3.2 Designs, integrates, and reworks digital content in a variety of formats. |  | 4.2. Understand intellectual property, copyright, and licensing as they relate to digital content. |  | b. The ability to use devices, technologies, techniques, and apps in research, quality improvement, audit, and scholarly activities. | Direct Patient Care, Administration, Informatics, Engineering/Information Systems/ICT, Research/Biomedicine | Participate in the selection, adoption, and utilization of appropriate health information technology tools |
|  |  | 3.3 Designs and fabricates objects using digital technology. |  | 4.3. Correctly use digital content creation tools to design and produce science and health content in digital environments, facilitating knowledge transfer and adding value (including for scientific research and publication). |  | c. The ability to use digital technologies to support or create new ideas, methods, solutions, and decisions. | Direct Patient Care, Administration, Informatics, Engineering/Information Systems/ICT, Research/Biomedicine | Evaluate the advantages and disadvantages of different electronic methods of obtaining and recording information in healthcare |
|  |  | 3.4 Programs. |  | 4.4. Acquire basic knowledge on how to design and produce digital content that promotes engagement with the intended audience (patients, public, other professionals, etc). | Change Management | d. The ability to act as a digital champion or change agent. |  |  |
|  |  |  |  | 4.5. Satisfy your individual creativity and solve everyday problems by creating and disseminating audience–appropriate digital content on the Internet. |  | e. The ability to lead on and champion the effective, appropriate, creative, and innovative use of digital technologies in research, scholarship, and other activities. |  |  |
| Collect, organize, analyze, interpret, and use data and data sets, including big data. (Data management and analytics) |  | 1.3 Analyzes, applies, and visualizes data using digital tools. | 6. Data analysis and management | 6.1. Know the main sources of health data and information in the field of healthcare. |  |  | Direct Patient Care, Administration, Informatics, Engineering/Information Systems/ICT, Research/Biomedicine | Use health information and ancillary software applications to create, run, and analyze reports, as pertinent to your role |
|  |  |  |  | 6.2. Possess the knowledge and skills necessary for advanced database management using common office automation tools. |  |  | Direct Patient Care, Administration | Construct and utilize appropriate inter and intraorganizational data exchange for better patient care (eHealth, health telematics applications, registry data exchange, etc.) |
|  |  |  |  | 6.3. Collect, organize, analyze, interpret, and use data and clinical information retrieved from different data sources. |  |  | Direct Patient Care, Administration, Informatics, Research/Biomedicine | Utilize and implement higher processes and functions of health information and support software, such as worksheets, charts, and graphs in order to achieve better data management |
|  |  |  |  | 6.4. Select or design optimal indicators for the analysis of health outcomes and internal processes in your area of expertise or clinical service. |  |  |  |  |
|  |  |  |  | 6.5. Extract knowledge from the large amounts of data (big data) generated in any clinical setting by applying the principles of evidence–based medicine. |  |  |  |  |
|  |  |  |  | 6.6. Understand the regulatory framework and the importance of security, privacy, and confidentiality of health data in the digital environment. |  |  |  |  |
| Drive and facilitate behavioral change in organizations and carry out process reengineering actions. (Change management) |  | 2.3 Gets involved in social and cultural change through the digital society. |  |  |  |  | Direct Patient Care, Administration, Informatics, Engineering/Information Systems/ICT, Research/Biomedicine | Contribute to the integration of health information exchanges and telehealth to improve care coordination between acute care and other providers, improve care transitions, increase access to specialist treatment, and support regional models of service delivery |
|  |  |  |  |  |  |  | Direct Patient Care | Encourage integration of telehealth into eHealth/health IT initiatives to empower patients with long-term conditions to manage their own condition, avoid unnecessary hospital admissions, provide support for both patients and caregivers, and provide greater access to specialist services |
| Identify and differentiate components, tools, and principles of ethics, reliability, and privacy in digital health. (Digital awareness) | Security, wellbeing, and civic mindedness | 4.1 Protects systems, devices, and digital content. | 1. Digital health literacy | 1.1. Be familiar with the devices, software programs, formats, document types, digital tools, and web platforms needed to perform daily work activities. | Digital Identity, Wellbeing, Safety, and Security | a. The ability to develop, promote and safeguard appropriate digital identity(-ies) that support a positive personal and organisational reputation. | Direct Patient Care | Develop and implement data governance based on your role and institution |
|  |  | 4.2 Protects personal data and privacy. |  | 1.2. Computer literacy: have the operational skills and basic proficiency to perform the computer activities necessary for daily work activities (web browsing, email management, use of word processors, spreadsheets, databases, information storage, etc). |  | b. The ability to use digital technologies in ways that support personal wellbeing and safety and the wellbeing and safety of others. | Direct Patient Care, Administration, Informatics, Engineering/Information Systems/ICT, Research/Biomedicine | Recognize the need to balance the privacy and security of health information and data with improved healthcare delivery, health technology development, and health system management |
|  |  | 4.3 Protects health, wellbeing, and the environment. |  | 1.3. Behave ethically and securely on the Internet (privacy, password management, etc), and be aware of and understand the potential risks associated with the Internet and electronic communication (email or web–based tools) in the workplace. |  | c. The ability to recognize and act upon digital situations and events that might compromise personal, professional, or organisational security. | Direct Patient Care | Develop decision support or alerts that can work in the medicines administration process with an eye for alert fatigue |
| Communication and digital reputation |  | 4.4 Manages digital identities and profiles. |  | 1.4. Identify, analyze, and critically review health websites in order to independently distinguish those that are reliable and of high quality from those that are not. |  | d. The ability to demonstrate and champion ethical, positive, healthy, and appropriate attitudes and behaviors in relation to digital identity, wellbeing, and safety of self and others. |  |  |
|  |  | 4.5 Behaves civically in the digital environment. |  |  |  | e. The ability to understand and manage the impact of own and others’ activities on the environment. |  |  |
|  | Autonomy and problem-solving | 5.1 Uses digital technology and understands its principles. |  |  | Technical Proficiency | a. The ability to use a wide range of technical devices in a personal and professional context both individually and with others. |  |  |
|  |  | 5.2 Identifies personal and professional needs and applies digital solutions. |  |  |  | b. The ability to use a wide range of software and apps for personal and professional use both individually and with others. |  |  |
|  |  | 5.3 Engages in digital skills updating. |  |  |  | c. The ability to resolve technical challenges and problems both individually and with others. |  |  |
|  |  | 5.4 Solves technical problems. |  |  |  | d. The ability to use technical knowledge to problem solve and achieve expected outputs. |  |  |
|  |  |  |  |  |  | e. The ability to support others with resolving technical challenges and problems and/or acting on technical opportunities. |  |  |
| Find, filter, select, monitor, store, and retrieve information online. (Information management) | Finding, managing, and analyzing information | 1.1 Finds, selects, and verifies information using digital tools. | 2. Efficient management of science and health information | 2.1. Know the channels through which digital information flows and learn to find, filter, select, monitor, evaluate, collate, store, organize, and retrieve it efficiently. | Information, Data, and Content | a. The ability to find, manage, organize, store, and share digital information, data, and content. | Direct Patient Care | Create, manage, and utilize policies for accessing, collecting, entering, retrieval, and storage of patient data, including single sign-on, remote record access, access pertaining to “scribes”, and other data access issues, as part of the appropriate care management team |
|  |  | 1.2 Organizes information and data using digital tools. |  | 2.2. Process information systematically and demonstrate a critical attitude that allows you to add value to information (content curation) and share it with the community. |  | b. The ability to understand and act upon appropriate guidelines, protocols, regulations, and safeguards in the use of differing media, information, data, and content to meet legal, ethical, cultural, and security rules, requirements, and expectations when working with personal, public, professional, and/or confidential information, data, and content. | Direct Patient Care, Administration, Informatics, Engineering/Information Systems/ICT | Facilitate meaningful discussions between clinical, administrative, and technical experts to ensure that health information and clinical records can be appropriately created, stored, aggregated, and retrieved |
|  |  | 1.3 Analyzes, applies, and visualizes data using digital tools. |  | 2.3. Recognize the criteria of quality, validity, and reliability of information available on the Internet and understand the legal and ethical principles that should govern the use of ICT. |  | c. The ability to critically analyse, evaluate and/or interpret information, data, content, and their sources. | Direct Patient Care, Administration | Interpret health information derived from individual data items |
|  |  |  |  |  |  | d. The ability to understand and adhere to digital copyright, intellectual property, and privacy rules and regulations. |  |  |
|  |  |  |  |  |  | e. The ability to work with and champion the effective, secure, appropriate, and innovative use of information, data and content in order to solve problems, make decisions, and to achieve successful outcomes for specific goals and objectives. |  |  |
| Professional development |  |  |  |  | Teaching, Learning, and Self-Development | a. The ability to use digital technologies and tools for personal learning and professional development. | Direct Patient Care | Facilitate the introduction of new systems or processes and support clinical colleagues during periods of change or modification |
|  |  |  |  |  |  | b. The ability to use a wide range of digital technologies and tools in teaching, coaching, and mentoring others. | Direct Patient Care, Administration, Informatics | Facilitate and promote patient/consumer use of health information and related technologies |
|  |  |  |  |  |  | c. The ability to demonstrate and champion a positive attitude in seeking out appropriate and innovative digital technologies to enhance learning for self and others. |  |  |
|  |  |  |  |  |  | d. The ability to design digital tools/resources/activities to support the teaching and learning of self and others. |  |  |
|  |  |  |  |  |  | e. The ability to manage/monitor the learning and development of self and/or others through digital technologies and tools. |  |  |
