# Supplementary figures and images for: Design, Implementation, and Analysis of an Assessment and Accreditation Model to Evaluate a Digital Competence Framework for Health Professionals: Mixed Methods Study
Source: JMIR Med Educ. 2024 Oct 17;10:e53462. doi: 10.2196/53462 (PMC11528169; doi:10.2196/53462)

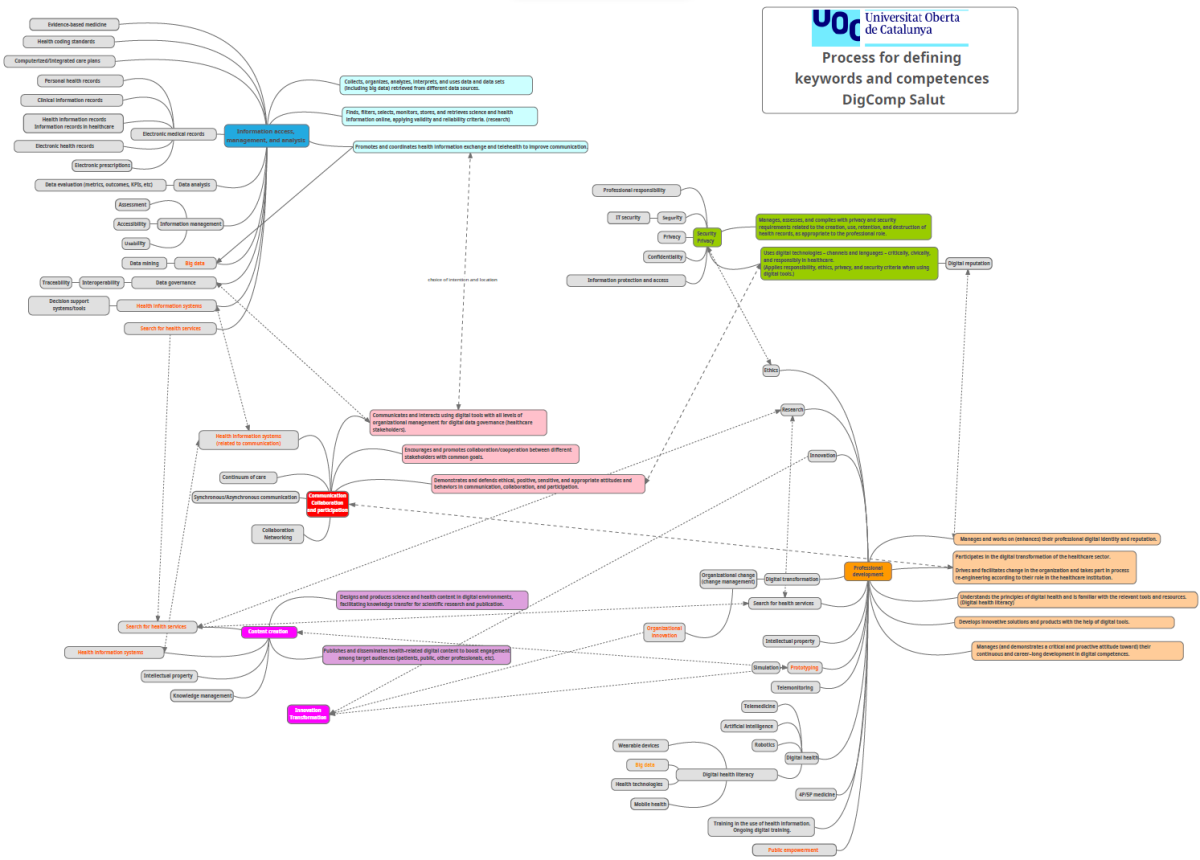

Supplement: Multimedia Appendix 3 [file mededu_v10i1e53462_app3.png]

**Appendix Figure 2.** Overall distribution of scores on Activity 1


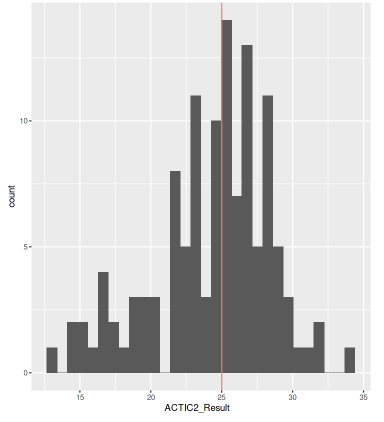

Supplement: Multimedia Appendix 8 [file mededu_v10i1e53462_app8.docx]
