# Supplementary material for: Design, Implementation, and Analysis of an Assessment and Accreditation Model to Evaluate a Digital Competence Framework for Health Professionals: Mixed Methods Study
Source: JMIR Med Educ. 2024 Oct 17;10:e53462. doi: 10.2196/53462 (PMC11528169; doi:10.2196/53462)
Supplement: Multimedia Appendix 4 [file mededu_v10i1e53462_app4.pdf]

Appendix 4. Web-based questionnaire 1

CompDig Health - Areas and Competencies Validation-GE

Greetings,

First of all, thank you for participating in this first validation of the proposed Areas and Competencies. To be able to carry out the validation, you can first take a loot at the presentation of the project:

1st Presentation of the project.
2nd Visual map that collects the competence areas, competencies and keywords.

Below, you will find a series of items that correspond to the competencies and a proposed competency area. Competency keywords are also defined to help you understand which concepts are included.

Keywords are those concepts that can be included in the achievement of competencies. As you will see, it does not refer to specific digital tools, as it is necessary to look for more general concepts that will remain valid in ever-changing digital work environments. These words can help you better contextualize the content of the competence.

The structure of the form is as follows:

Firstly, Area 1 is presented with its description and later appear the skills that need to be validated. Subsequently, Area 2 appears and so on until area 4. In order to validate the competencies, three ASPECTS must be taken into account:

- The FORMULATION, that is to say, the wording is clear, concrete and with appropriate language.
- The COHERENCE, that is to say, the competence is consistent with the corresponding area.
- The APPLICABILITY and RELEVANCE, that is to say, the competence is important and applicable to health professions.

You will need to mark with an X one of the following 4 options for each of the aspects:

- Yes, completely.
- Yes, moderately.
- Yes, in some aspects.
- Does not comply

At the end of each area, you will find a box where you can provide comments or observations about the competencies and keywords of the area.

*A time commitment of approximately 30 minutes to 1 hour is expected.

*Mandatory

Name and Surname*

Professional Profile*

Competence area 1 - Access, Management and Data Analysis

Just as or even more important than knowing how and where to search is to verify the information, compare it, and select the information that interests us, meets our objectives, and is truthful.

On a daily basis, a large volume of data is introduced, consulted and analyzed from different sources of information, such as the Electronic Clinical History, departmental systems, communication systems... Managing this large volume of information that we have at hand has become a necessary skill in order to organize it and retrieve it effectively when needed.

Analyzing this information and the data we manage will be very beneficial for generating new useful information that will benefit us and assist in making assessments and decisions.

Competence 1.1: Manages healthcare data and information, in any of its phases (collection, monitoring, storage, recovery, filtering, destruction...) coming from different sources of information and formats.

KEYWORDS: Health coding standards, Integrated systems (care continuum), Health and social information registers, Information management, Data governance: Traceability and Interoperability, Health information systems, Decision support systems / Decision support tools, Search for Health services.

Mark only one oval per row.

	Yes, completely	Yes, moderately	Yes, in some respects	Does not comply
Formulation	◯	◯	◯	◯
Coherence	◯	◯	◯	◯
Applicability and relevance	◯	◯	◯	◯

Observations of competence 1.1

Competence 1.2: Performs analysis and interpretation of data and datasets with the help of digital tools, algorithms, AI and big data.

KEY WORDS: Health coding standards, Integrated systems (care continuum), Health and social information registers, Information management, Data governance: Traceability and

Interoperability, Health information systems, Decision support systems / Decision support tools, Search for Health services.

Mark only one oval per row.

	Yes, completely	Yes, moderately	Yes, in some respects	Does not comply
Formulation	◯	◯	◯	◯
Coherence	◯	◯	◯	◯
Applicability and relevance	◯	◯	◯	◯

Observations of competence 1.2

Competence 1.3: Analyzes and interprets data and data sets with the help of digital tools, algorithms, AI and massive data.

KEY WORDS: Health coding standards, Integrated systems (care continuum), Health and social information registers, Information management, Data governance: Traceability and Interoperability, Health information systems, Decision support systems / Decision support tools, Search for Health services.

Mark only one oval per row.

	Yes, completely	Yes, moderately	Yes, in some respects	Does not comply
Formulation	◯	◯	◯	◯
Coherence	◯	◯	◯	◯
Applicability and relevance	◯	◯	◯	◯

Observations of competence 1.3

Regarding Area 1 Access, Management and Data Analysis, have you found any competencies/topics/keywords missing?

Mark only one oval

- ◯ Yes
- ◯ No

If you have selected Yes, please indicate below what have you missed. You can add other observations and comments.

Competence area 2 - Communication and collaboration

Thanks to technology, sending messages, audios, images or videos to exchange information, express thoughts and ideas has become an everyday activity, both in the professional and personal fields, which allows us to:

- Interact with different individuals involved (patients, other professionals, citizens...), communicate and exchange data between different clinical-care information systems in a transparent and safe way for the professional.
- Collaborate with other people, develop projects and create resources in a team within a digital environment; and participate in this communicative environment to express ourselves, give opinions and get involved in health and social development.
- Create and disseminate digital content by adapting the channels and languages to the target audience and the context.

Competence 2.1: Promotes communication, interaction and exchange of healthcare information and data through digital tools (synchronous/asynchronous), adapted to the different agents involved.

KEY WORDS: Health information systems, Synchronous/asynchronous communication, Digital care-citizen relationship.

Mark only one oval per row.

	Yes, completely	Yes, moderately	Yes, in some respects	Does not comply
Formulation	◯	◯	◯	◯
Coherence	◯	◯	◯	◯
Applicability and relevance	◯	◯	◯	◯

Observations of competence 2.1

Competence 2.2: Enhances and promotes network collaboration between different agents with shared goals and objectives.

KEY WORDS: Information systems in Health, Synchronous/asynchronous communication, digital healthcare-citizen relationship, collaborative work in network.

Mark only one oval per row.

	Yes, completely	Yes, moderately	Yes, in some respects	Does not comply
Formulation	◯	◯	◯	◯
Coherence	◯	◯	◯	◯
Applicability and relevance	◯	◯	◯	◯

Observations of competence 2.2

Competence 2.3: Create, publish and disseminate digital content related to health, assessing the context and the most appropriate channel and taking into account the target and the intended recipients. (citizens, patients, healthcare professionals or external).

KEY WORDS: Information systems in Health, Synchronous/asynchronous communication, digital healthcare-citizen relationship, collaborative work in network.

Mark only one oval per row.

	Yes, completely	Yes, moderately	Yes, in some respects	Does not comply
Formulation	◯	◯	◯	◯
Coherence	◯	◯	◯	◯
Applicability and relevance	◯	◯	◯	◯

Observations of competence 2.3

Regarding Area 2 about communication and collaboration, have you found any competencies/topics/keywords missing?

Mark only one oval

○ Yes

○ No

If you have selected Yes, please indicate below what have you missed. You can add other observations and comments.

Competence area 3 - Digital awareness

It is essential to carry out the professional activities in a digital environment in a safe and civic way. To ensure the well-being of everyone in the digital health field we need to know skills, regulations and attitudes that help us protect applications and devices, data, and take care of privacy.

Likewise, we must guarantee sufficient knowledge to protect the intellectual property of the applications developed and the prototypes designed for a healthcare and/or social organization.

Competence 3.1.: Ensures protocol compliance, regulatory frameworks and regulations on privacy, confidentiality, and the protection of health information and data and evaluates them.

KEYWORDS: Digital reputation, Security, Privacy, Confidentiality, Protection and Access to information, Ethics, Intellectual Property.

Mark only one oval per row.

	Yes, completely	Yes, moderately	Yes, in some respects	Does not comply
Formulation	◯	◯	◯	◯
Coherence	◯	◯	◯	◯
Applicability and relevance	◯	◯	◯	◯

Observations of competence 3.1

Competence 3.2: Applies criteria of responsibility, safety and civility in the use of digital technologies - channels, tools and languages - in digital health.

KEYWORDS: Digital reputation, Security, Privacy, Confidentiality, Protection and access to information, Ethics, Intellectual Property.

Mark only one oval per row.

	Yes, completely	Yes, moderately	Yes, in some respects	Does not comply
Formulation	◯	◯	◯	◯
Coherence	◯	◯	◯	◯
Applicability and relevance	◯	◯	◯	◯

Observations of competence 3.2.

Competence 3.3: Demonstrates and advocates for ethical, sensitive, and appropriate attitudes and behaviors in the healthcare digital environment.

KEYWORDS: Digital reputation, Security, Privacy, Confidentiality, Protection and access to information, Ethics, Intellectual Property.

Mark only one oval per row.

	Yes, completely	Yes, moderately	Yes, in some respects	Does not comply
Formulation	◯	◯	◯	◯
Coherence	◯	◯	◯	◯
Applicability and relevance	◯	◯	◯	◯

Observations of competence 3.3

Regarding Area 3 about Digital awareness, have you found any competence/topic/keyword missing?

Mark only one oval

- ◯ Yes
- ◯ No

If you have selected Yes, please indicate below what have you missed. You can add other observations and comments.

Competence area 4 - Professional development

Once the fundamentals and basic functionalities of digital health technology have been achieved, we can develop our autonomy in a progressive manner. It's also time to work on our professional identity to maintain a positive reputation towards our healthcare work.

It is essential that healthcare professionals incorporate digital foundations with concepts, languages, channels, and technologies linked to digital health and that they stay updated throughout their professional lives; to be able to act as agents of change and participate in the digital transformation through innovative projects, telemedicine, mobility...

Competence 4.1.: Manage your professional identity and ensure a positive digital reputation.

KEYWORDS: Digital reputation, Professional digital identity, Ethics.

Mark only one oval per row.

	Yes, completely	Yes, moderately	Yes, in some respects	Does not comply
Formulation	◯	◯	◯	◯
Coherence	◯	◯	◯	◯
Applicability and relevance	◯	◯	◯	◯

Observations of competence 4.1

Competence 4.2: Participate and promote digital transformation in the healthcare field.

KEY WORDS: Research, Innovation, Digital transformation, Organizational change (change management).

Mark only one oval per row.

	Yes, completely	Yes, moderately	Yes, in some respects	Does not comply
Formulation	◯	◯	◯	◯
Coherence	◯	◯	◯	◯
Applicability and relevance	◯	◯	◯	◯

Observations of competence 4.2

Competence 4.3: Understands the fundamentals of digital health - tools, devices and resources - and shows an active attitude towards constant digital learning and updating.

KEYWORDS: Literacy in digital health.

Mark only one oval per row.

	Yes, completely	Yes, moderately	Yes, in some respects	Does not comply
Formulation	◯	◯	◯	◯
Coherence	◯	◯	◯	◯
Applicability and relevance	◯	◯	◯	◯

Observations of competence 4.3

Regarding area 4 about Professional development, have you found any competences/topics/keywords missing?

Mark only one oval

◯ Yes

◯ No

If you have selected Yes, please indicate below what have you missed. You can add other observations and comments.

Other comments (on the name of the areas, the overall competences, or any idea or observation).
