# Supplementary material for: Design, Implementation, and Analysis of an Assessment and Accreditation Model to Evaluate a Digital Competence Framework for Health Professionals: Mixed Methods Study
Source: JMIR Med Educ. 2024 Oct 17;10:e53462. doi: 10.2196/53462 (PMC11528169; doi:10.2196/53462)
Supplement: Multimedia Appendix 5 [file mededu_v10i1e53462_app5.pdf]

Appendix 5. Web-based questionnaire 2

COMPDIG-Health - Areas, Competencies and Indicators Validation- GRFDP

Greetings,

First of all, thank you for your participation in this competency and indicator validation form, which have been defined by the accreditation model of Specific Digital Competencies for healthcare professionals of Catalonia.

To be able to carry out the validation, you can preview the attached summary table that contains the areas, competencies and indicators.

The structure of the following form is as follows:

Each section is a Competence Area where the competences are included with the corresponding indicators (two or three per competence).

In order to validate the competencies and indicators, three ASPECTS must be taken into account:

- The FORMULATION, that is to say, the wording is clear, concrete and with appropriate language.
- The COHERENCE, that is to say, the competence is consistent with the corresponding area.
- The APPLICABILITY and RELEVANCE, that is to say, the competence is important and applicable to health professions.

You will need to mark with an X one of the following 4 options for each of the aspects:

- Yes, completely
- Yes, moderately
- Yes, in some aspects
- Does not comply

At the end of each area, you will find a box where you can provide comments or observations about the competencies and indicators of the area.

*A time commitment of approximately 1 hour is expected.

* Mandatory

Competence area: Access, management and data analysis

Just as or even more important than knowing how and where to search is to verify the information, compare it, and select the information that interests us, meets our objectives, and is truthful.

On a daily basis, a large volume of data is introduced, consulted and analyzed from different sources of information, such as the Electronic Clinical History, departmental systems, communication systems... Managing this large volume of information that we have at hand has become a necessary skill in order to organize it and retrieve it effectively when needed.

Analyzing this information and the data we manage will be very beneficial for generating new useful information that will benefit us and assist in making assessments and decisions.

Competence 1 (Data Management). Manages healthcare and social data and information, in any of its phases (access, collection, monitoring, storage, recovery, filtering, elimination...) coming from different sources of information and formats. *

	Yes, completely	Yes, moderately	Yes, in some respects	Does not comply
Formulation	◯	◯	◯	◯
Coherence	◯	◯	◯	◯
Applicability and relevance	◯	◯	◯	◯

About the indicators of Competence 1: *

Indicators
1.1 Uses the different sources of healthcare and social information data that are most appropriate depending on the pursued objective.<br><br>1.2. Ensures that the information linked to the healthcare and social registry reflects suitability, quality, integrity, and authenticity.<br><br>1.3. Applies and promotes organizational and regulatory policies and measures for access, collection, monitoring, storage, retrieval, filtering, and disposal of healthcare and social data and information according to the professional role.

	Yes, completely	Yes, moderately	Yes, in some respects	Does not comply
Formulation	◯	◯	◯	◯
Coherence	◯	◯	◯	◯
Applicability and relevance	◯	◯	◯	◯

Competence 1 and Indicators: Observations and comments (include the number of the indicator you want to comment on).

Competence 2 (Data analysis). Analyze and interpret data and data sets with the help of digital tools, algorithms, artificial intelligence and big data. *

	Yes, completely	Yes, moderately	Yes, in some respects	Does not comply
Formulation	◯	◯	◯	◯
Coherence	◯	◯	◯	◯
Applicability and relevance	◯	◯	◯	◯

About the indicators of Competence 2: *

Indicators
2.1. Obtains data from websites, services and/or applications and processes them to facilitate decision-making in the healthcare and social environment (dashboards, business intelligence tools, web analytics, ...).
2.2. Organizes and synthesizes data to create, execute, and analyze reports according to the professional role.
2.3. Selects and designs optimal indicators to measure, compare, group and relate the data according to the purpose.

	Yes, completely	Yes, moderately	Yes, in some respects	Does not comply
Formulation	○	○	○	○
Coherence	○	○	○	○
Applicability and relevance	○	○	○	○

Competence 2 and Indicators: Observations and comments (include the number of the indicator you want to comment on).

Competence area: Communication and collaboration

Thanks to technology, sending messages, audios, images or videos to exchange information, express thoughts and ideas has become an everyday activity, both in the professional and personal fields, which allows us to:

- Interact with different individuals involved (patients, other professionals, citizens...), communicate and exchange data between different clinical-care information systems in a transparent and safe way for the professional.
- Collaborate with other people, develop projects and create resources in a team within a digital environment; and participate in this communicative environment to express ourselves, give opinions and get involved in health and social development.
- Create and disseminate digital content by adapting the channels and languages to the target audience and the context.

Competence 3 (Communication). Encourages communication, interaction and the exchange of health and social information and data through digital tools (synchronous/asynchronous) and adapted to the different actors involved.

	Yes, completely	Yes, moderately	Yes, in some respects	Does not comply
Formulation	○	○	○	○
Coherence	○	○	○	○
Applicability and relevance	○	○	○	○

About the indicators of Competence 3: *

Indicators
3.1. Encourage the use of digital health communication tools to facilitate assistance.
3.2. Chooses and uses the most appropriate digital communication tool according to the context and the actors involved in the different services available.
3.3. Supports the different actors in the use of tools to exchange health and social data.

	Yes, completely	Yes, moderately	Yes, in some respects	Does not comply
Formulation	◯	◯	◯	◯
Coherence	◯	◯	◯	◯
Applicability and relevance	◯	◯	◯	◯

Competence 3 and indicators: Observations and comments (include the number of the indicator you want to comment on).

Competence 4 (Collaboration). Enhances and promotes network collaboration among different actors with shared goals and objectives.

	Yes, completely	Yes, moderately	Yes, in some respects	Does not comply
Formulation	◯	◯	◯	◯
Coherence	◯	◯	◯	◯
Applicability and relevance	◯	◯	◯	◯

About the indicators of Competence 4: *

Indicators
4.1. Promotes the incorporation of digital tools to innovate or improve collaboration and teamwork methodologies in the health and social teamwork.<br><br>4.2. Detects and actively participates in different networks linked to the health and social field, both at national and international level.<br><br>4.3. Promotes connections between different individuals and organizations in networks related to the healthcare and social field for the exchange of learning, knowledge, resources, and best practices

	Yes, completely	Yes, moderately	Yes, in some respects	Does not comply
Formulation	◯	◯	◯	◯
Coherence	◯	◯	◯	◯
Applicability and relevance	◯	◯	◯	◯

Competence 4 and indicators: Observations and comments (include the number of the indicator you want to comment on).

Competence 5 (Digital contents). Create, publish and share digital content related to health, assessing the context and the most appropriate channel and aiming the target and recipients (citizens, users of Health Services, health professionals or external). *

	Yes, completely	Yes, moderately	Yes, in some respects	Does not comply
Formulation	◯	◯	◯	◯
Coherence	◯	◯	◯	◯
Applicability and relevance	◯	◯	◯	◯

About the indicators of Competence 5: *

Indicators
5.1. Uses digital design and editing tools to produce scientific-health content adapted to the target audience and the communication channel.<br><br>5.2. Defines a digital publication and dissemination strategy to achieve the defined goals according to the professional role.

	Yes, completely	Yes, moderately	Yes, in some respects	Does not comply
Formulation	◯	◯	◯	◯
Coherence	◯	◯	◯	◯
Applicability and relevance	◯	◯	◯	◯

Competence 5 indicators: Observations and comments (include the number of the indicator you want to comment on).

Competence area: Digital awareness

It is essential to carry out the professional activities in a digital environment in a safe and civic way. To ensure the well-being of everyone in the digital health field we need to know skills, regulations and attitudes that help us protect applications and devices, data, and take care of privacy.

Likewise, we must guarantee sufficient knowledge to protect the intellectual property of the applications developed and the prototypes designed for a healthcare and/or social organization.

Competence 6 (Data protection). Ensures compliance with protocols, regulatory frameworks and regulations on privacy, confidentiality and protection of health and social information and data. *

	Yes, completely	Yes, moderately	Yes, in some respects	Does not comply
Formulation	◯	◯	◯	◯
Coherence	◯	◯	◯	◯
Applicability and relevance	◯	◯	◯	◯

About the indicators of Competence 6: *

Indicators
6.1. Applies the current requirements about privacy, confidentiality and protection of health data information.
6.2. Critically evaluates privacy and security requirements related to the records and disclosure of protected health and social information, according to the professional role.
6.3. Reports any potential breach, improper retention or destruction of health information, and ensures that appropriate corrective actions are taken when the privacy or security of any confidential information has been compromised.

	Yes, completely	Yes, moderately	Yes, in some respects	Does not comply
Formulation	○	○	○	○
Coherence	○	○	○	○
Applicability and relevance	○	○	○	○

Competence 6 and indicators: Observations and comments (include the number of the indicator you want to comment on).

Competence 7 (Ethics and civility). Applies ethical principles, security criteria, and civility in the responsible use of digital technologies - channels, tools, and languages - in healthcare. *

	Yes, completely	Yes, moderately	Yes, in some respects	Does not comply
Formulation	○	○	○	○
Coherence	○	○	○	○
Applicability and relevance	○	○	○	○

About the indicators of Competence 7: *

Indicators
7.1. Uses mechanisms to maintain the security of systems and devices in the exchange of health and social information.
7.2. Respects copyright and use intellectual property licenses when designing, implementing, researching and/or disseminating health and social content.
7.3. Promotes and guarantees best practices of respect, coexistence and ethics in the health and social digital environment.

	Yes, completely	Yes, moderately	Yes, in some respects	Does not comply
Formulation	◯	◯	◯	◯
Coherence	◯	◯	◯	◯
Applicability and relevance	◯	◯	◯	◯

Competence 7 and indicators: Observations and comments (include the number of the indicator you want to comment on).

Competence area: Professional development

Once the fundamentals and basic functionalities of digital health technology have been achieved, we can develop our autonomy in a progressive manner. It's also time to work on our professional identity to maintain a positive reputation towards our healthcare work.

It is essential that healthcare professionals incorporate digital foundations with concepts, languages, channels, and technologies linked to digital health and that they stay updated throughout their professional lives; to be able to act as agents of change and participate in the digital transformation through innovative projects, telemedicine, mobility...

Competence 8 (Digital literacy). He/She is constantly training and actualizing about digital health. *

	Yes, completely	Yes, moderately	Yes, in some respects	Does not comply
Formulation	○	○	○	○
Coherence	○	○	○	○
Applicability and relevance	○	○	○	○

About the indicators of competence 8: *

Indicators
8.1 Shows a critical and proactive attitude towards learning in digital competence throughout professional life.<br><br>8.2. Identifies the most advanced technologies and assesses their possibilities in the healthcare field.

	Yes, completely	Yes, moderately	Yes, in some respects	Does not comply
Formulation	○	○	○	○
Coherence	○	○	○	○
Applicability and relevance	○	○	○	○

Competence 8 and indicators: Observations and comments (include the number of the indicator you want to comment on).

Competence 9 (digital identity). Manage his/her professional digital identity and looks after a digital reputation. *

	Yes, completely	Yes, moderately	Yes, in some respects	Does not comply
Formulation	◯	◯	◯	◯
Coherence	◯	◯	◯	◯
Applicability and relevance	◯	◯	◯	◯

About the indicators of Competence 9: *

Indicators
9.1 Protects professional digital identity and differences from personal digital identity.
9.2 Monitors and applies strategies to optimize his/her professional digital reputation.

	Yes, completely	Yes, moderately	Yes, in some respects	Does not comply
Formulation	◯	◯	◯	◯
Coherence	◯	◯	◯	◯
Applicability and relevance	◯	◯	◯	◯

Competence 9 and indicators: Observation and comments (include the number of the indicator you want to comment on).

Competence 10 (Digital transformation). Participates and promotes digital transformation in the healthcare sector. *

	Yes, completely	Yes, moderately	Yes, in some respects	Does not comply
Formulation	◯	◯	◯	◯
Coherence	◯	◯	◯	◯
Applicability and relevance	◯	◯	◯	◯

About the indicators of Competence 10: *

Indicators
**10.1 Takes part in the redefinition of digital transformation processes according to their role in the institution.**<br><br>**10.2 Innovates in the generation of digital solutions and/or products in the field of health and social care according to the professional role.**<br><br>**10.3 Participate actively and constructively in the debate on digital transformation in the health and social sector.**

	Yes, completely	Yes, moderately	Yes, in some respects	Does not comply
Formulation	◯	◯	◯	◯
Coherence	◯	◯	◯	◯
Applicability and relevance	◯	◯	◯	◯

Competence 10 and indicators: Observations and comments (include the number of the indicator you want to comment on).

General comments and observations
