# Supplementary material for: Design, Implementation, and Analysis of an Assessment and Accreditation Model to Evaluate a Digital Competence Framework for Health Professionals: Mixed Methods Study
Source: JMIR Med Educ. 2024 Oct 17;10:e53462. doi: 10.2196/53462 (PMC11528169; doi:10.2196/53462)
Supplement: Multimedia Appendix 6 [file mededu_v10i1e53462_app6.pdf]

Appendix 6. Information sheet and recruitment questionnaire

Recruitment - Piloting the model for the assessment of digital competencies in healthcare

Disclaimer: This form is intended for digitally competent healthcare professionals*.

The Professional Dialogue Forum identified 17 present and future challenges within the framework of the Catalan health system. One of the challenges identified is the need for improving the digital skills of healthcare professionals (Challenge 4) and the "COMPDIG-Salut" project is carrying out its implementation.

The "COMPDIG-Salut" project has developed a digital skills assessment model for healthcare professionals (PPS). This model has been built based on the specific digital competencies map for PPS, which has been previously validated by the Challenge 4 Group of the Professional Dialogue Forum. It is therefore necessary to validate the model in a controlled environment and with the participation of professionals with advanced digital skills.

If you agree to participate, the following form will collect your name, surname, and email, among other questions, to recruit you for the pilot test. Within a maximum of 48 hours, you will receive an email to the provided address that will give you access to a Moodle environment. In this environment, you will find three quizzes: a level test (based on ACTIC-2), a digital competencies assessment test, and an assessment questionnaire. You can respond until March 31. For the analysis, the data will be anonymized.

The estimated total time to complete the three questionnaires is 1.5 hours, but they can be completed independently within the period of the study (1 month). Your answers will be used to evaluate the designed evaluation model. You can leave the study at any time.

You have until March 31st to participate in this study. Thank you for your interest!

The project is led by the "Fundació TIC Salut Social" in collaboration with the General Direction of Health Professionals of the Department of Health, the Professional Management of CatSalut and the Department of the Vice-Presidency and of Digital Policies and Territory.

For more information and resolution of doubts about the study you can contact the following email: COMPDIG.SALUT@TICSALUTSOCIAL.CAT

*Healthcare professions according to the definition of Law 44/2003 of November 21: Biologist specialist, Dietitian-Nutritionist, Pharmacist, Physicist-Chemist specialist.

* Mandatory

Information on data protection

Responsible for the treatment: Fundació TIC Salut Social, with NIF G64350374, address at Street Roc Boronat, 81-95, 08005 Barcelona and contact of the data protection officer at dpd-FTSS@ticsalutsocial.cat

Purpose: Project management and, specifically, the ability to assess the model for evaluating digital competencies of healthcare professionals (Activity 2) in order to advance towards its implementation.

Rights of data subjects: You can request access, rectification, deletion or limitation and portability of the processing of your data, as well as revoke consent at any time, by contacting dpd-FTSS@ticsalutsocial.cat. In case of disagreement, you can file a complaint with the Catalan Data Protection Authority (www.apdcat.cat).

For more information, you can consult the "Projectes" processing activity in the Processing Activities Register at https://ticsalutsocial.cat/transparencia

1. I authorize Fundació TIC Salut Social to process the information I provide, including my personal data, for the purpose indicated, including in specific circumstances, send communications to the email I provide to confirm some of my responses in this form. *

   ○ Yes

   ○ No

2. Additionally, I confirm that I am interested in participating voluntarily in the described pilot test and that I am aware that I can withdraw my participation at any time. *

   ○ Yes

   ○ No

Participant information collection

Enter your first and last name. *

Enter your email address. *

Indicate what your healthcare profession is: *

- ◯ Specialist biologist
- ◯ Dietitian-Nutritionist
- ◯ Pharmacist
- ◯ Physicist /Chemist specialist
- ◯ Physiotherapist
- ◯ Dental hygienist
- ◯ Nurse
- ◯ Speech therapist
- ◯ Doctor
- ◯ Dentist
- ◯ Optician Optometrist
- ◯ Podiatrist
- ◯ Dental protein
- ◯ Clinical or health phycologist
- ◯ Occupational therapist
- ◯ Others

What is your self-perception of your level in digital skills? *

- ◯ **Advanced user level** (I have achieved the most advanced digital skills to transform and innovate today's digital society. I promote digital projects and I provide guidance to others in the attainment of digital competencies).
- ◯ **Average user level** (I have an active role in the digital ecosystem (I participate, create and disseminate content). I use the advanced features of technological tools and applications, and I enjoy autonomy and critical capacity towards the use of digital technologies).

Have you taught any day or session related to digital skills applied to the field of health? *

(Select one or more answers)

- ☐ Yes, as a speaker and/or trainer
- ☐ Yes, as an organizer
- ☐

No

Do you have any digital skills certification, such as ACTIC? *
(If multiple answers apply, select the one with the highest grading)

- Yes, ACTIC basic level
- Yes, ACTIC medium level
- Yes, ACTIC advanced level
- No, I don't have any
- Others

What is your professional profile? *
(Check the option that best fits your current professional work)

- **Direct patient-contact healthcare** (Professionals who dedicate more than 70% of their working day to providing assistance or service to patients directly. Medical staff, nurse, occupational therapist, speech therapist, optician-optometrist, dental hygienist, dental practitioner, pharmacist, etc.).
- **Non-direct patient-contact healthcare** (Professionals who spend more than 70% of their working day supporting care services. Medical staff in the biological diagnosis and pathology services. Specialist biologist staff, specialist in physics and chemistry, pharmacist, dental prosthetics, etc.).
- **Innovation, research and teaching** (professionals who dedicate more than 70% to provide services in the fields of innovation, research and/or teaching. Examples: researchers, innovation technicians, etc.).
- **Management** (professionals who devote more than 70% to the management of entities, departments, services or work teams. Examples: managers I intermediate commands).
