# Supplementary material for: Design, Implementation, and Analysis of an Assessment and Accreditation Model to Evaluate a Digital Competence Framework for Health Professionals: Mixed Methods Study
Source: JMIR Med Educ. 2024 Oct 17;10:e53462. doi: 10.2196/53462 (PMC11528169; doi:10.2196/53462)
Supplement: Multimedia Appendix 7 [file mededu_v10i1e53462_app7.docx]

**Table 2.** Specific map of digital competences for health professionals.

| Competence area and keywords | | Competences | Indicators |
| --- | --- | --- | --- |
| **Data access, management, and analysis** | | | |
|  | - Integrated systems (continuum of care) - Health and social records - Information management - Data governance—traceability and interoperability - Health information systems - Decision support systems or tools - Health service research - Health service evaluation | - Data management   - Manages health and social data and information at all stages (eg, access, collection, monitoring, storage, retrieval, filtering, and deletion) from different sources and in different formats. | - Uses the most appropriate sources of health and social data for the intended purpose. - Ensures that the information in health and social records is appropriate, of high quality, complete, and authentic. - Applies and promotes organizational and regulatory policies and measures for accessing, collecting, monitoring, storing, retrieving, filtering, and deleting health and social data and information according to their role. |
|  | - Health coding standards (eg, CIM^a^, ATIC^b^, NIC^c^/NOC^d^/NANDA^e^, and SNOMED^f^) - Data analysis and evaluation (eg, metrics, outcomes, and KPIs^g^) - Big data | - Data analysis   - Analyzes and interprets data and data sets using algorithms, artificial intelligence, big data, and digital tools. | - Retrieves data from websites, services, or applications and processes them to facilitate decision-making in health and social care settings (eg, dashboards, business intelligence tools, and web analytics). - Organizes and synthesizes data to create, run, and analyze reports as pertinent to their role. - Selects and designs optimal indicators to measure, compare, group, and correlate data according to purpose. |
| **Communication and collaboration** | | | |
|  | - Health information systems - Synchronous and asynchronous communication - Digital relationship between health care and the public | - Communication   - Fosters communication, interaction, and exchange of health and social data and information using digital tools (synchronous and asynchronous) as appropriate for those involved. | - Promotes the use of digital health communication tools to facilitate care, including telecare. - Selects and uses the most appropriate digital communication tools considering the context and the people involved in the given service. - Assists others in using tools to exchange health and social data. |
|  | - Collaborative networking | - Collaboration   - Strengthens and promotes collaborative networking among people with common goals. | - Promotes the use of digital tools to innovate or improve collaboration and teamwork practices in health and social care settings. - Identifies and actively participates in national and international networks related to health and social care. - Connects people and organizations in health and social care networks to share experiences, knowledge, resources, and best practices. |
|  | - Health information systems - Digital relationship between health care and the public | - Digital content   - Creates, publishes, and shares health-related digital content considering the context and the best channel for the intended purpose and audience (public, health care users, health professionals, or external professionals). | - Uses digital design and editing tools to produce science and health content tailored to the target audience and communication channel. - Defines digital publication and dissemination strategies to achieve specific goals according to their professional role. |
| **Digital awareness** | | | |
|  | - Security - Privacy - Confidentiality - Information protection and access | - Data protection   - Ensures compliance with protocols, legal frameworks, and regulations regarding privacy, confidentiality, and protection of health and social data and information. | - Adheres to current requirements for privacy, confidentiality, and protection of health and social data. - Critically reviews privacy and security requirements related to the recording and disclosure of protected health and social information according to their professional role. - Reports or denounces potential breaches or wrongful withholding or destruction of health and social information and ensures that appropriate corrective action is taken when the privacy or security of confidential information has been compromised. |
|  | - Digital reputation - Ethics - Intellectual property - Code of ethics | - Ethics and civic-mindedness   - Adheres to ethical principles, applies security criteria, and demonstrates civic behavior in the responsible use of digital health technologies (ie, channels, tools, and languages). | - Uses mechanisms to maintain the security of systems and devices in the exchange of health and social information. - Respects copyright and uses intellectual property licenses when designing, implementing, researching, or disseminating health and social content. - Promotes and ensures respectful, civic, and ethical behavior in digital health and social environments. |
| **Professional development** | | | |
|  | - Digital health literacy | - Digital training   - Continually seeks education and training in digital health. | - Demonstrates a critical and proactive attitude toward career-long development in digital competences. - Identifies the most advanced technologies and considers their potential in health care. |
|  | - Professional digital identity - Personal digital identity | - Digital identity   - Manages their professional digital identity and ensures an optimal digital reputation. | - Protects their professional digital identity and keeps it separate from their personal digital identity. - Monitors and implements strategies to optimize their professional digital reputation. |
|  | - Digital transformation | - Digital transformation   - Participates in and promotes digital transformation in health care. | - Participates in the definition or redefinition of digital transformation processes according to their institutional role. - Identifies and prioritizes the development of digital solutions or products in health and social care according to their professional role. - Participates actively and constructively in the debate on digital transformation in health and social care. |

^a^CIM: International Classification of Diseases

^b^ATIC: ATIC: Architecture, Terminology, Interface, Information, Nursing (Infermeria), and Knowledge (Coneixement)

^c^NIC: Nursing Interventions Classification

^d^NOC: Nursing Outcomes Classification

^e^NANDA: North American Nursing Diagnosis Association

^f^SNOMED: Systematized Nomenclature of Medicine

^g^KIP: Key Performance Indicator
