# Supplementary material for: Design, Implementation, and Analysis of an Assessment and Accreditation Model to Evaluate a Digital Competence Framework for Health Professionals: Mixed Methods Study
Source: JMIR Med Educ. 2024 Oct 17;10:e53462. doi: 10.2196/53462 (PMC11528169; doi:10.2196/53462)

**Appendix Figure 3.** Overall distribution of scores in the proposed assessment and accreditation test for health professionals


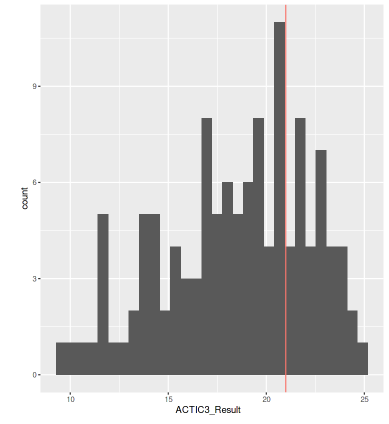

Supplement: Multimedia Appendix 9 [file mededu_v10i1e53462_app9.docx]
