# Supplementary material for: Design, Implementation, and Analysis of an Assessment and Accreditation Model to Evaluate a Digital Competence Framework for Health Professionals: Mixed Methods Study
Source: JMIR Med Educ. 2024 Oct 17;10:e53462. doi: 10.2196/53462 (PMC11528169; doi:10.2196/53462)
Supplement: Multimedia Appendix 10 [file mededu_v10i1e53462_app10.docx]

**Appendix Table 3.** Discrimination indexes (DI) of the instrument items for profile P1

| **Challenge** | **Item** | **DI** | **Interpretation** |
| --- | --- | --- | --- |
| 1 | 1 | -0.067 | Needs review |
| 1 | 2 | 0.175 |  |
| 1 | 3 | -0.030 | Needs review |
| 1 | 4 | 0.285 | Good discriminator |
| 1 | 5 | 0.420 | Good discriminator |
| 1 | 6 | 0.205 | Good discriminator |
| 1 | 7 | 0.082 |  |
| 1 | 8 | 0.179 |  |
| 1 | 9 | 0.236 | Good discriminator |
| 2 | 10 | 0.170 |  |
| 2 | 11 | 0.180 |  |
| 2 | 12 | 0.115 |  |
| 2 | 13 | 0.212 | Good discriminator |
| 2 | 14 | 0.262 | Good discriminator |
| 2 | 15 | 0.330 | Good discriminator |
| 2 | 16 | 0.214 | Good discriminator |
| 2 | 17 | 0.418 | Good discriminator |
| 2 | 18 | 0.350 | Good discriminator |
| 2 | 19 | 0.166 |  |
| 2 | 20 | 0.106 |  |
| 2 | 21 | 0.164 |  |
| 2 | 22 | 0.252 | Good discriminator |
| 2 | 23 | 0.283 | Good discriminator |
| 2 | 24 | 0.266 | Good discriminator |
| 2 | 25 | 0.410 | Good discriminator |
| 2 | 26 | 0.381 | Good discriminator |
| 2 | 27 | 0.112 |  |
| 2 | 28 | 0.177 |  |
