# Supplementary material for: Design, Implementation, and Analysis of an Assessment and Accreditation Model to Evaluate a Digital Competence Framework for Health Professionals: Mixed Methods Study
Source: JMIR Med Educ. 2024 Oct 17;10:e53462. doi: 10.2196/53462 (PMC11528169; doi:10.2196/53462)
Supplement: Multimedia Appendix 11 [file mededu_v10i1e53462_app11.docx]

**Appendix Table 4.** Question 5, ‘Feedback on the profile–specific challenges and scenarios’

| **Categories** | **n** |  | **%** |
| --- | --- | --- | --- |
| Positive feedback | 26 |  | 27.7 |
| Does not fully identify with the proposed challenges and scenarios | 33 |  | 35.1 |
| Does not identify with the proposed challenges and scenarios | 12 |  | 12.8 |
| Other | 16 |  | 17.0 |
| Total contributions | 94 |  | 100.0 |
