# Supplementary material for: Design, Implementation, and Analysis of an Assessment and Accreditation Model to Evaluate a Digital Competence Framework for Health Professionals: Mixed Methods Study
Source: JMIR Med Educ. 2024 Oct 17;10:e53462. doi: 10.2196/53462 (PMC11528169; doi:10.2196/53462)
Supplement: Multimedia Appendix 12 [file mededu_v10i1e53462_app12.docx]

**Appendix Table 5.** Question 5, ‘Feedback on the profile–specific challenges and scenarios by profession’

| **Categories** | **n** | **%** |
| --- | --- | --- |
| Positive feedback  Nurses and physicians  Other professions | 26  11  15 | 27.7  42.3  57.7 |
| Does not fully identify with the proposed challenges and scenarios  Nurses and physicians  Other professions | 33  11  22 | 35.1  33.3  66.7 |
| Does not identify with the proposed challenges and scenarios  Nurses and physicians  Other professions | 12  1  11 | 12.8  8.3  91.7 |
| Other | 16 | 17.0 |
| Total contributions | 94 | 100.0 |
