# Supplementary material for: Design, Implementation, and Analysis of an Assessment and Accreditation Model to Evaluate a Digital Competence Framework for Health Professionals: Mixed Methods Study
Source: JMIR Med Educ. 2024 Oct 17;10:e53462. doi: 10.2196/53462 (PMC11528169; doi:10.2196/53462)
Supplement: Multimedia Appendix 13 [file mededu_v10i1e53462_app13.docx]

**Appendix Table 6.** Question 7, ‘Feedback on the profiles’

| **Categories** | **n** | **%** |
| --- | --- | --- |
| Positive feedback | 49 | 62.0 |
| Does not agree with the defined percentages | 5 | 6.3 |
| Does not fully agree with the 4 defined profiles | 7 | 8.9 |
| Other | 18 | 22.8 |
| Total contributions | 79 | 100.0 |
