# Supplementary material for: Design, Implementation, and Analysis of an Assessment and Accreditation Model to Evaluate a Digital Competence Framework for Health Professionals: Mixed Methods Study
Source: JMIR Med Educ. 2024 Oct 17;10:e53462. doi: 10.2196/53462 (PMC11528169; doi:10.2196/53462)
Supplement: Multimedia Appendix 14 [file mededu_v10i1e53462_app14.pdf]

Appendix 14. Example Scenario 1.

Accreditation CompDIG_Salut - Scenario 1

General aspects of the test

- Incorrect answers are not deducted.
- It is necessary to pass 70% of the final mark.
- It should be noted that in multiple-choice questions, incorrect answers subtract 20% of the score. No marks are deducted for simple answers (single answers).
- Question type underlined in yellow.
- Positive and negative scores in bold next to each answer.
- Select the random mix option for your answers.

Profile P1. Direct patient care
Professionals who spend more than 70% of their workday providing direct patient care or services. Physicians, nurses, occupational therapists, speech therapists, optometrists/opticians, dental hygienists, pharmacists, etc.

CONTEXT: You are a professional in a healthcare organization providing care in the outpatient setting.

28 questions = 30 marks. 21 points are required to achieve a pass mark.

CHALLENGE: YOU ARE IN YOUR CLINICAL OFFICE WHERE YOU ATTEND TO YOUR PATIENTS

A new patient arrives from another centre. As this requires creating a new medical record in your centre, do you inform the user about the organization's policies related to data management and processing?
(Multiple-choice question: only one answer)

A. No. This is not my job.
B. No. The patient has no rights over his or her data, as it is health information.
C. Yes, I inform my patients about how the Electronic Health Record works and how to exercise their rights. (1 point)
D. No. Everyone should be responsible for their data and, if users are not interested, it is not necessary to inform them.

One of the patients you visited had a complication related to Maxillofacial Surgery. Your centre's service portfolio does not include this specialty, so you consider it appropriate to contact an acquaintance in this specialty who works at the referral hospital in your health area. You call him and he asks you to share clinical information about the patient. How do you share the information?
(Multiple choice question: multiple answers)

A. By WhatsApp, as it offers immediacy and allows you to share images and files of the patient's clinical process. You consider it a secure system because the servers where the WhatsApp service is hosted are located in the European Union. (-20%)
B. By Telegram, because it offers the same immediacy as WhatsApp and has a greater security system, which allows you to share images and files of the patient's clinical process. (-20%)
C. By e-mail, attaching, in a first message, the patient's clinical documentation in a compressed and password-protected file and, in a second e-mail message, the password to access the file. (0,5 points)
D. By a corporate instant messaging system (such as XatSalut, MedXat, etc.) that guarantees the security of the information shared. (0.5 points)

You receive a 58-year-old patient on a first visit with a chronic pathology who has a surgical history of a programmed intervention carried out in your centre. When you want to start writing the clinical course of the patient, you copy the surgical history. You review the content and detect some errors. How do you act?
(Multiple choice question: only one answer)

A. You contact the doctor responsible for the patient to inform him/her that there are erroneous data. (0.5 points)
B. You delete the copied and pasted content and start the clinical course again.
C. You modify the text you have entered, putting now the correct dates. (0.5 points)
D. Options a and c are correct. (1 point)

The next visit is a 28-year-old patient who is normally seen by your colleague in the department and who is now on sick leave. When you consult his medical history, you notice that there is a piece of information in the case history that is coded as Eisenmenger's syndrome. Before the patient enters the consulting room, you want to look at some bibliographic references related to the pathology. You access your organization's bibliographic resources search engine to look for literature associated with the disease. How would you search for updated results in the last twelve months?
(Drag and drop into an image, see image)
Drag the appropriate option into the search box and the word selects into the appropriate search options.

A. Eisen
B. Eisen*
C. "Eisen*"
D. Syndrome AND Einsen*. (move to a red box 0,50 points)
E. Syndrome *Eisen
F. Eisen -Syndrome
G. Eisen-
H. Activate filter. (move to the box marked yellow - 0,5 points)

Image

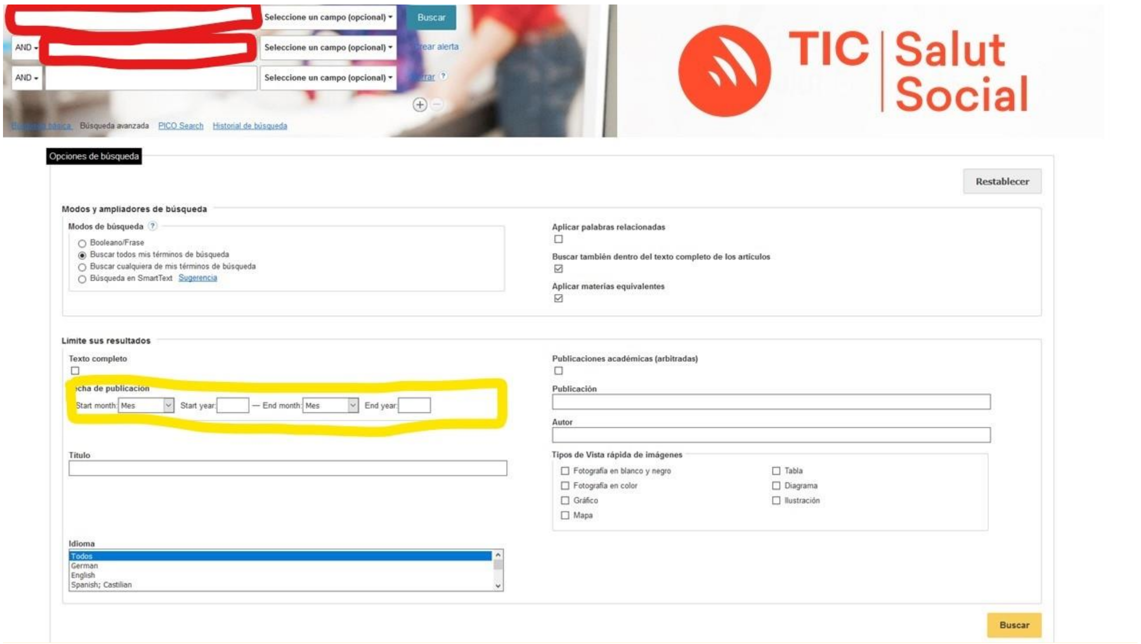

You have seen several patients, and you have to code the procedures and diagnoses of each of the attendances or episodes. Do you know how coding relates to other processes or circuits?

(Multiple choice question: multiple answers)

A. It makes interoperability with external applications impossible. (-20%)
B. Facilitates interoperability with internal applications only. (-20%)
C. Facilitates interoperability with internal and external applications. (0,3 points)
D. Enables the exploitation of information. (0.4 points)
E. It prevents large volumes of data from being collected. (-20%)
F. It is essential for invoicing. (0.3 points)
G. It is an indispensable requirement to make a referral to another service. (-20%)

You receive a call from a patient asking you to update the end date of a prescription for a medication for a chronic condition. He is a middle-aged patient. He asks you to provide him with a prescription and that he will come and pick it up. What is your response?

(Multiple choice question: only one answer)

A. You confirm that you will prescribe for him to come and pick it up.
B. You confirm that you can write her a prescription and that she can find it on the My Health app. (1 point)
C. You confirm that you wrote the prescription for him/her to come and pick it up and that he/she has the updated medication plan in the My Health application. (0.3 points)
D. You confirm that you prescribe because the prescription for medicines and medical devices is not published in My Health or in the Shared Health Record of Catalonia (HC3) due to the confidentiality of patient data.

If you visit a patient who needs to be scheduled for a particular service, what would you do to improve the process of claiming the service?
(Multiple choice question: only one answer)

A. Schedule the benefit from the patient's My Health.
B. Although this is a request that requires clinical judgment, the request could be made using an assisted conversation bot.
C. The process could be automated using natural language processing systems in the Electronic Health Record. (1 point)
D. All options are appropriate.

You are visiting a 78-year-old man who lives in a nursing home in the same municipality as your centre. He has mobility difficulties and, as no associated diagnostic tests are scheduled for the next follow-up visits, you consider it appropriate to carry out the following visits by seeing the patient telematically. How will you carry out these visits?
(Multiple choice question: only one answer)

A. With a WhatsApp video call, so that the patient can send you the clinical information you request during the visit.
B. With Microsoft Teams, as it is a videoconferencing tool widely used by Catalan health and social organizations.
C. With the corporate videoconferencing or telemedicine tool. (1 point)
D. Using a telephone call because neither video nor message sharing is necessary.
E. using a free tool that you have searched for on the Internet and that requires the installation of an executable file, both on the device of the sender of the message and on the device of the receiver.

The last patient you visited today tells you that he has shared his case with a childhood friend who is a specialist in his pathology and has done so using an e-mail message providing personal and sensitive data. How do you deal with this situation?
(Multiple choice question: only one answer)

A. There is no problem because I tell him/her to delete the message sent.
B. There is no problem, as long as no data linked to the healthcare professional attending to the patient appears.
C. I provide the patient with an infographic on data protection that we have prepared in our Service. (1 point)
D. Personal data can be included in e-mail messages because it is a peer-to-peer technology that does not allow data to be obtained on the content of messages sent and received.

CHALLENGE: YOU ARE WORKING IN THE ROOM YOU SHARE WITH THE COLLEAGUES OF YOUR MEDICAL SERVICE

When you check your e-mail inbox, you read a message (you are in copy) in which a colleague from your department consults with a colleague from another specialty about doubts regarding the request for complementary tests for a hospitalized patient. The consultation is made using an e-mail message and providing the patient's details. How do you act in this situation?

(Multiple choice question: only one answer)

A. There is no problem, as the e-mail sent is then deleted if necessary.
B. I comment, privately, that patient data should not be included in this type of consultation. (1 point)
C. I reply to the message asking not to be copied as this is not my patient.
D. Personal data can be included by e-mail because all e-mails are automatically encrypted.

A colleague tells you that she would like to access the medical records of a patient you are treating because he is her neighbor and has asked her to write a report for his GP. What is your answer?

(Multiple choice question: only one answer)

A. OK, since the health data belongs to the health professionals, who are the agents entering the data into the organization's medical records.
B. Requires special permission, given that the health or social organization, apart from guaranteeing the custody of the Health Record, is the owner of the data.
C. Cannot access, as the medical record belongs exclusively to the patient. (1 point)
D. No problem, given that the health data belong to the Health System, and this is evident with the appearance of the Shared Health Record in Catalonia.

Today, the team members have set about defining which indicators they will use to manage the department more effectively. One of the team members suggests that in order to be able to make algorithmic predictions of the service's care activity, it would be interesting to obtain data prospectively, and not only retrospectively. Which technologies do you think could help you?

(Multiple choice question: multiple answers)

A. Augmented Reality. (-20%)
B. Virtual Reality. (-20%)
C. Big data. (0.33 points)
D. Artificial intelligence. (0.33 points)
E. Business intelligence (business intelligence). (-20%)
F. Internet of things. (-20%)
G. Assisted conversation boats. (-20%)

H. Collaborative platforms. (-20%)
I. Social and professional networks. (-20%)
J. Cloud computing. (0.34 points)

For the definition of the indicators that will help the effective management of the Service you choose...

(Multiple choice question: multiple answers)

A. You ask the Information Systems or Information Management service of your organization to define them. (-20%)
B. Visits made. (0.11 points)
C. First visits. (0.11 points)
D. Return to the ED in less than 48 hours. (-20%)
E. Successive visits. (0.11 points)
F. Repetition rate. (0.11 points)
G. Hospital stays. (-20%)
H. Scheduling time. (0.11 points)
I. Duration of visits. (0.12 points)
J. Number of total visits. (0.11 points)
K. Pharmaceutical expenditure. (0.11 points)
L. Waiting time. (0.11 points)
M. Surgical re-interventions. (-20%)
N. Indicators for other services or specialties. (-20%)

Once you have defined the indicators, you want to produce a report on the results. What tools can you use to perform each of the tasks?

(Drag and drop into a text)

A. I will analyze the data with (0.16 points) ……. or with (0.17 points) …….
B. I will write a basic report document with (0.16 points) ……. or with (0.17 points) …….
C. Finally, I will produce an infographic with (0.17 points) ……. or (0.17 points) …….

Piktochart
Wiki
monday.com
Mailchimp
Vismo (group c)
Google Analytics (group a)
Canva (grupo bic)
OpenOffice Writer (group b)
miro.com
Trello
Genially (group c)
Google Docs (groups a, b)

Infogram (group c)
R (grupo a)
Google Forms
Stata (grupo a)
LucidChart
OpenOffice Calc (group a)
Kanban (group c)
BaseCamp
Slack
TikTok
Asana

The Care Management explains that the organization is starting a corporate Digital Transformation project and asks for your opinion. During question time you ask for the floor and comment:
(Multiple choice question: only one answer)

A. You think that the Digital Transformation Plan only affects the organization's Information Systems Management and, therefore, you think that it should not actively collaborate.
B. You think that it is a good opportunity to participate in the analysis and redefinition, if necessary, of the organizational circuits and processes in which you are involved and which can be improved. (1 point)
C. You think that this is a task in which only an external consultancy firm should be involved.
D. You consider that it is a process that contributes nothing to the organization, as it adapts to changes continuously without the need to draw up a Digital Transformation Plan.

You have detected that different patients in the Department feel anxious in the moments prior to undergoing a diagnostic test. Taking advantage of the visit by the Healthcare Management on the occasion of the Digital Transformation Plan, a colleague indicated that there is evidence of the usefulness of different technological devices to reduce this anxiety. What type of technology do you think would be the most appropriate?
(Multiple choice question: only one answer)

A. Big data, showing the patient that, after analyzing thousands of data from different diagnostic tests, it can be shown that 98% of these tests have no side effects.
B. Virtual Reality, showing how the service staff works with a recording where the facilities and the procedure can be visualized in an immersive way. (1 point)
C. With an artificial intelligence tool and the creation of an algorithm capable of predicting in detail how the diagnostic test will be carried out.
D. With the inclusion of a patient traceability tool, which will allow relatives and companions to know where the patient is at any given moment and when the diagnostic test has started and finished.

Taking advantage of the fact that the Healthcare Management is sharing the future Digital Transformation project of the organization with the entire Department, you agree that it would be interesting to incorporate different integrations with the Electronic Health Record. What integrations would you propose?
(Multiple choice question: only one answer)

A. Integrations with departmental applications from external providers, such as Diagnostic Imaging solutions (0,5 points)
B. Electromedical devices, such as electrocardiograms, ultrasound scanners, spirometers... (0.5 points)
C. In the Electronic Health Record, data can only be entered by the organization's healthcare professionals and not by data from other systems.

D. I would choose options a and b. (1 point)

In chronic pathologies, patients often find it difficult to follow the assigned treatments correctly. In the case of some pathologies, it is easy for them to abandon them and forget about regular check-ups. Given this problem, what would you suggest?
(Multiple choice question: only one answer)

A. If the pathology allows it, you suggest integrating data obtained by the patient himself in everyday situations through wearable devices (wearables) in the records of his Electronic Health Record. (1 point)
B. In order to be more effective and improve follow-up, you ask the patient to send you the data/measurements that the wearable devices record on a continuous basis and thus have all the data available.
C. Given that online consultations do not help to improve adherence to treatment and monitoring of the disease, you suggest increasing face-to-face follow-up visits.
D. Current medical history systems do not allow for the incorporation of external data, and you prefer not to propose anything.

After discussing the integration of patient wearables into the Electronic Health Record, the Management proposes to create a virtual Community of Practice to explain this integration by sharing it with our residents and trainees, as well as with specialists from other health and social organizations. What do you think?
(Multiple choice question: multiple answers)
A. You think we should not share this good practice to avoid plagiarism by other organizations. (-20%)
B. You think that the virtual Community of Practice should be created to promote the exchange of learning, knowledge, and resources. (0.5 points)
C. Apart from creating a virtual Community of Clinical Practice, you think that the case of integration should also be shared in different social networks. (0.5 points)
D. You think that this good practice should not be shared because of an issue related to data protection. (-20%)

In terms of the best practices presented in the virtual Community of Practice, how will we protect the intellectual property of the content?
(Multiple choice question: only one answer)

A. As we will either make the content ourselves or commission the organization's Communication Department to do so, ownership is automatically attributed to the organization.
B. There is no need to take anything into account, as this is health education material and therefore not-for-profit content.
C. We will use Creative Commons licenses. (1 point)
D. If we show the authorship of the contents of the material, it is enough to guarantee the intellectual property.

To disseminate different publications of the Service among the population served by the organization, which digital spaces would you use?
List the tools you would use depending on the content and age group.
(Matching question. List the tools and you must choose one of the 3 options) Total score 2 points.

- 1. Carry out a health education campaign linked to the prevention of a prevalent pathology in your department using an infographic aimed at an age group between 18-30 years old.
- 2. A 2-minute health promotion video linked to the Mediterranean diet as prevention of pathologies diagnosed and treated by your department, aimed at a population between 30 and 55 years of age.
- 3. A 15-slide presentation showing the results of the indicators of the Service's report by residents and other trainees.

Twitter (1, 2 and 3)
Facebook (2)
Instagram (1, 2)
LinkedIn (3)
YouTube (2)
Tik Tok (1)
SlidePlayer (3)
SlideShare (3)

You have decided that the contents resulting from the best practices presented in the virtual Community of Clinical Practice should be shared on social networks. Under which profile do you comment on or publish these contents?
(Multiple choice question: only one answer)

A. I post from my personal profile, as I don't have a professional profile because I don't usually post anything work-related.
B. I post from both profiles I have (personal and professional), so the more "likes" I get, the better it will be to promote the publication.
C. I don't have social networks and it should only be published from a corporate account of the organization.
D. From my professional profile, because I differentiate between personal publications and those related to my profession (1 point).

Do you want to check if there are similar experiences shared in networks and communities? Where would you look? 2 points
(Drag within a text)
Choose the search spaces or tools and fill in the boxes with the corresponding tools.

- 1 Social network 0.25 points add three boxes.

- 2 Meta search engines social networks 0,25 points add two boxes.
- 3 Internet search engines 0,25 points add two boxes.
- 4 RSS readers 0,25 points add two boxes.
- 5 Public specialized portals (highest score) 0,5 points add three boxes.
- 6 Private specialized portals (highest score) 0.5 points add three boxes.

Twitter (1)
LinkedIn (1)
Instagram (1)
Social Searcher (2)
TalkWalker (2)
Google Trends (3)
BuzzSumo (3)
Feedly (4)
Flipboard (4)
Aquas (5)
ICT Health and Social Foundation (5)
Department of Health (5)
Digital Health Association (6)
Catalan Society of Digital Health (6)
Professional Associations (6)

In order to improve the way of working online with the rest of the team members and with the residents and trainees, you propose to the head of the service to incorporate an online collaborative tool. What tools would you propose?
(Multiple choice question: multiple answers)

A. We will use Microsoft Teams because it is the corporate tool. (0.5 points)
B. You are thinking of collaborative tools such as Microsoft OneDrive, Slack, Monday.com... (0,5 points)
C. We will work with files located on the network, because if a user is working with a file, another user who wants to connect to the same file can make a copy and, therefore, both users can work with the same file at the same time. (-20%)
D. We are unable to share online documentation with residents and trainees due to data confidentiality issues. (-20%)

A collaborative cloud environment has been created, and you need to access it to make an amendment to a confidential document in the centre. How do you connect?
(Multiple choice question: only one answer)

A. From your personal mobile phone using the school's public Wi-Fi network.
B. It makes no difference how I connect, as the data is in the cloud and is password protected.
C. From any device in the organization with a secure connection and up-to-date antivirus. (1 point)

D. The b y c options are suitable.

The health service you are working for is designing and publishing health education and health promotion content aimed at the general public. How do you design this material?
(Multiple-choice question: only one answer)

A. Do you use the same material for all target groups, regardless of age, cultural level, etc.?
B. You are creating material with a lot of text and little graphics because you want to explain in detail the message you want to convey.
C. You are designing very graphic material with older patients in mind and video format for the younger group. (1 point)
D. The b y c options are appropriate.

Fill in the text:
(Select the missing words)

A. Training in big data is being organized. The head of service considers that you should be trained in this area.
B. You already know what big data is (drop-down A).
C. What would be your position on this topic and training?
D. Well, (drop-down B) I would do the training because I think that (Drop-down C)

Drop-down A: definition of big data and others:
- A set of data which, due to its volume, nature, and the speed at which it must be processed, is beyond the capacity of the usual computer systems. (0.3 points)
- Large and complex dataset that originates only from data sources outside the organization.
- Storage of large amounts of information data, whether internal or external, which do not need to be analyzed.

Drop-down B:
- Yes (0,3 points)
- No
- Don't know if

Deployable C:
- Not useful for our day-to-day life.
- We only need occasional training when a particular application or device changes.
- It is part of the organization’s digital training plan and has therefore already been assessed for its suitability. (0.4 points)
- I am open to changes, and they can always give us an idea. (0.1 points)

In order to promote telemedicine in your Service, you want to define and guarantee a series of good practices. In order to define them, it is necessary to take them into account: *(Multiple choice question: multiple answers)*

A. Distance healthcare is not governed by the same legal and ethical principles as face-to-face healthcare. (-20%).
B. Telemedicine, as a complement to face-to-face care, has no legal framework. (-20%)
C. Distance healthcare is governed by the same legal and ethical principles that govern face-to-face healthcare. (0.5 points)
D. Confidentiality and security of data must be ensured from data collection to data management, storage, and transfer between centres. (0.5 points)
